# Low-frequency magnetic fields do not aggravate disease in mouse models of Alzheimer's disease and amyotrophic lateral sclerosis

**DOI:** 10.1038/srep08585

**Published:** 2015-02-26

**Authors:** Martina P. Liebl, Johannes Windschmitt, Anna S. Besemer, Anne-Kathrin Schäfer, Helmut Reber, Christian Behl, Albrecht M. Clement

**Affiliations:** 1 Institute for Pathobiochemistry, University Medical Center of the Johannes Gutenberg-University, Mainz, Germany; 2 Department of Nuclear Medicine, University Medical Center of the Johannes Gutenberg-University, Mainz, Germany

## Abstract

Low-frequency magnetic fields (LF-MF) generated by power lines represent a potential environmental health risk and are classified as possibly carcinogenic by the World Health Organization. Epidemiological studies indicate that LF-MF might propagate neurodegenerative diseases like Alzheimer's disease (AD) or amyotrophic lateral sclerosis (ALS). We conducted a comprehensive analysis to determine whether long-term exposure to LF-MF (50 Hz, 1 mT) interferes with disease development in established mouse models for AD and ALS, namely APP23 mice and mice expressing mutant Cu/Zn-superoxide dismutase (SOD1), respectively. Exposure for 16 months did not aggravate learning deficit of APP23 mice. Likewise, disease onset and survival of SOD1^G85R^ or SOD1^G93A^ mice were not altered upon LF-MF exposure for ten or eight months, respectively. These results and an extended biochemical analysis of protein aggregation, glial activation and levels of toxic protein species suggests that LF-MF do not affect cellular processes involved in the pathogenesis of AD or ALS.

The etiology of age-related, progressive neurodegenerative diseases like Alzheimer's disease (AD) and amyotrophic lateral sclerosis (ALS) is largely unknown. Less than ten percent of patients show a familial history of disease indicating that the vast majority of patients develop AD and ALS for so far unknown reasons. AD and ALS are both detrimental diseases that affect different neuronal cell populations. In AD primarily neurons in the cortex and the hippocampus degenerate, whereas primary and secondary motor neurons of the motor cortex, the brain stem and the spinal cord are prominently affected in ALS. AD and ALS also differ in the disease course. In contrast to ALS that typically is a fast progressing disease diagnosed between 40 and 60 years, patients suffering from sporadic forms of AD develop first symptoms even later in life and the disease course is progressive over decades. Besides genetic predisposition and the presence of certain genetic polymorphisms, age-related alterations of the metabolism as well as environmental factors are believed to contribute to the initiation of AD and ALS^1^,^2^. Epidemiological studies and subsequent meta-analysis indicate that the exposure to low frequency magnetic fields (LF-MF; 50 Hz) might be one of the potential risk factors to develop these disorders^3^,^4^.

The exposure to LF-MF that are generated by power lines and the use of electrical devices has constantly increased with technical progress. There is a growing public interest in potential effects of LF-MF exposure on human health because epidemiological studies and subsequent meta-analyses associate occupational as well as residential LF-MF exposure with disease conditions like childhood leukemia, AD, and ALS^3^,^4^,^5^. These data prompted the Environmental Health Criteria Report 238 by the World Health Organization (WHO) on electromagnetic fields and public health^6^ and the categorization of LF-MF into the group 2B as “possibly carcinogenic to humans”^7^. It is nevertheless uncertain if and how the exposure to LF-MF might affect functions of the human brain like pain perception, memory formation, motor control, and sleep^8^.

It is controversially discussed how LF-MF might affect cellular function on the molecular level in general and whether disease-associated cellular pathways are affected. Opposing results generated in several experimental studies might be caused by the use of different model systems and different LF-MF exposure paradigms. For example, the complex discussion regarding the potential effect of electromagnetic field exposure on the oxidative status of cells and tissues is extensively reviewed by Consales and colleagues^9^. To our knowledge, no comprehensive study has been conducted so far to investigate the impact of long-term exposure to LF-MF on the initiation and the progression of AD and ALS in adequate animal models under controlled laboratory conditions.

To investigate whether long-term exposure to LF-MF has an impact on pathways affected in AD and ALS, we continuously exposed well-accepted genetic mouse models of both human diseases to LF-MF (50 Hz, 1 mT). The magnetic flux density of 1 mT was about tenfold above the guidelines of the German Federal Immission Control Act^10^ and the European Council recommendation^11^ for resident exposures. APP23 mice that overexpress the Swedish double mutation of the amyloid precursor protein (APP) under control of the murine Thy-1 promoter develop pathological hallmarks like Aβ plaques and acquire learning deficits^12^. Two transgenic mouse lines expressing mutant variants of the Cu/Zn-superoxide dismutase (SOD1), namely SOD1^G85R^ and SOD1^G93A^, develop a progressive phenotype in adult mice resulting in a prominent loss of motor neurons and finally a complete paralysis^13^,^14^. Here we show that based on behavioral and life span assessment and on the extended biochemical analysis of protein aggregation, glial activation and levels of toxic proteins, long-term exposure to LF-MF of mouse models for AD and ALS did not aggravate the disease course.

## Results

### APP and Aβ levels of APP23 mice are not altered upon LF-MF exposure

**Figure 1:**
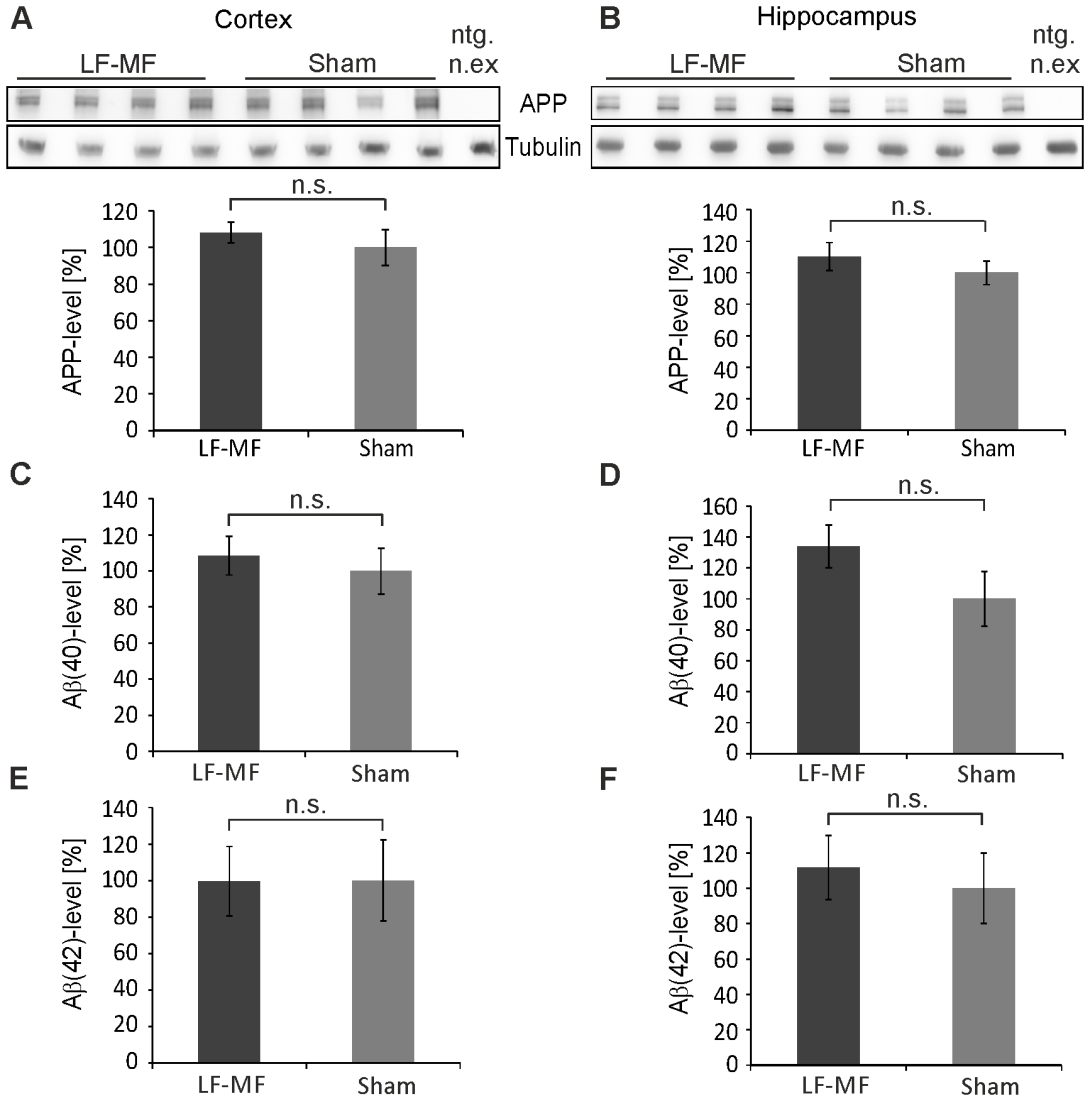
APP, Aβ_(40)_ and Aβ_(42)_ levels in APP23 mice are not altered upon LF-MF exposure. (A, B) Lysates from cortices (A) and hippocampi (B) of 18 month old female APP23 mice were separated by SDS-PAGE and transferred onto nitrocellulose membranes. APP was detected with specific antibodies (upper band) and band intensities were determined. Tubulin served as a loading control and protein levels were normalized to that of sham exposed animals. Antibodies against APP and tubulin were consecutively applied and developed on the same membrane (Supplemental Fig. 9) (mean +/− SEM; n = 11 (cortex) and n = 10 (hippocampus) for LF-MF, n = 13 for sham; Mann-Whitney-U-test; n.s. not significant). (C–F) Aβ_(40)_ (C, D) and Aβ_(42)_ (E, F) levels in the cortex (C, E) and the hippocampus (D, F) were determined by ELISA and levels were normalized to sham exposed animals (mean +/− SEM; cortex: Aβ_(40)_ LF-MF n = 10, sham n = 8; Aβ_(42)_ LF-MF n = 10, sham n = 9; hippocampus: Aβ_(40)_ and Aβ_(42)_ LF-MF n = 10, sham n = 9; Mann-Whitney-U-Test, n.s. not significant).

After genotyping, APP23 mice were exposed continuously to a magnetic field from about 2 months to 18 months of age. Aged APP23 mice develop a prominent learning deficit and are characterized by depositions of Aβ-plaques in the brain^12^. Human APP and Aβ levels were monitored as increased levels of mutant APP correlate with a more severe pathological phenotype^15^. We quantitatively determined total human APP and soluble Aβ_(40)_ and Aβ_(42)_ levels by Western blot and Aβ-subtype-specific ELISA, respectively. Both, total APP levels as well as soluble Aβ_(40)_ and Aβ_(42)_ levels were unaffected upon exposure condition in the cortex and the hippocampus of female (Fig. 1) and male mice (not shown). In line with these data, the number of Aβ-plaques was unchanged upon exposure in the frontal, medial, and caudal regions of the cortex and the hippocampus (Supplemental Fig. 1). In addition, we observed in the cortex and the hippocampus no statistically significant differences in sAPPα and sAPPβ levels, representing the extracellular APP-fragments after the non-amyloidogenic and amyloidogenic processing of APP, respectively (Supplemental Fig. 2). These data indicate that the long-term exposure of APP23 mice to LF-MF did not affect the protein levels and processing of APP.

### Glial activation and LF-MF exposure in APP23 mice

**Figure 2:**
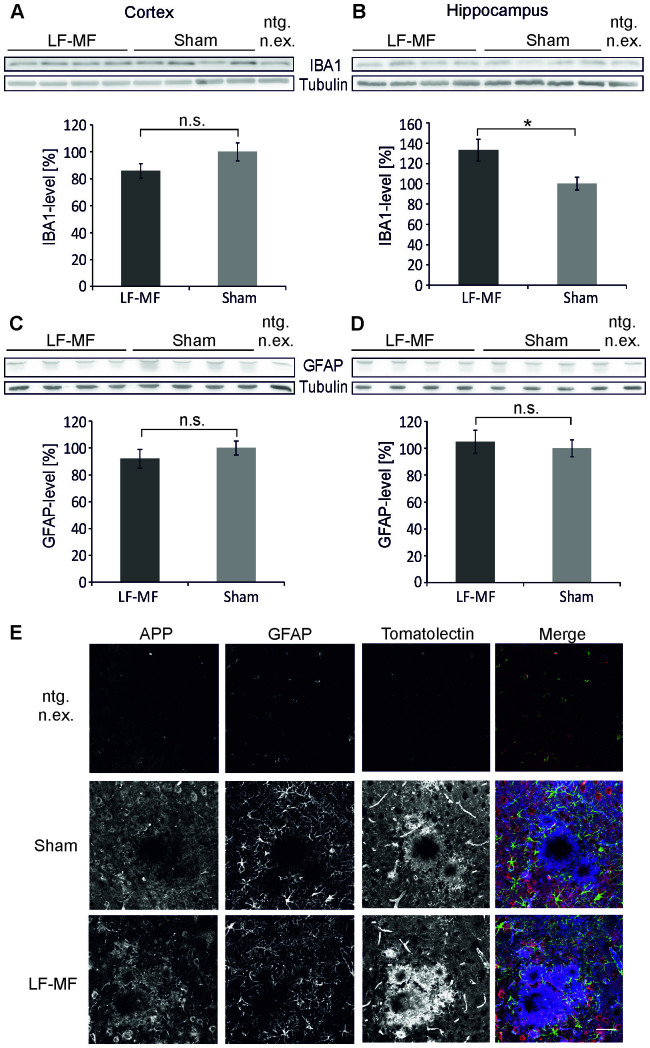
IBA1 and GFAP expression is comparable in LF-MF and sham exposed APP23 mice. (A–D) Protein levels of IBA1 (A, B) and GFAP (C, D) in the cortex (A, C) and hippocampus (B, D) of 18 month old female APP23 mice were determined by Western Blot. The densitometric analysis is shown in corresponding lower panels. Tubulin served as a loading control and data were normalized to sham exposed animals. IBA1 and tubulin antibodies or GFAP and tubulin antibodies were applied and developed consecutively on the same membrane (Supplemental Fig. 11) (mean +/− SEM; IBA1: cortex n = 11 for LF-MF and sham; Mann-Whitney-U-Test; hippocampus LF-MF n = 10, sham n = 12: t-test; GFAP: cortex: n = 11 for LF-MF and sham; hippocampus LF-MF n = 10, sham n = 12; Mann-Whitney-U-Test; * p<0.05). (E) Confocal pictures of coronal sections of brains from 18 month old exposed and sham exposed APP23 mice and non-transgenic littermates (ntg. n.ex.). Sections were stained with antibodies against APP (red) and GFAP (green) and corresponding secondary antibodies. Microglia were detected with tomatolectin conjugated to Alexa488 (blue) (bar: 50 µm).

Microglia activation and astrocytosis are pathological hallmarks of Alzheimer's disease. Recent evidence suggests that microglia activation is not a secondary effect of tissue damage, but plays a pivotal role in mediating Aβ-toxicity^16^. We therefore analyzed the levels of IBA1 and glial fibrillary acidic protein (GFAP) to monitor activation of microglia and astrocytes, respectively. IBA1 protein levels were increased in the hippocampus (Fig. 2B) but not in the cortex (Fig. 2A) of female APP23 mice after exposure and in male tissues (not shown). GFAP-levels were not different in the cortex and the hippocampus of female (Fig. 2C–D) and male mice (not shown) upon exposure to LF-MF. Similar results were obtained when IBA1 and GFAP expression were analyzed by immunofluorescent stainings of the affected tissues (Fig. 2E). These data indicate that exposure to LF-MF did not generally affect microglia activation.

### Spatial learning is not affected in APP23 mice upon exposure

**Figure 3:**
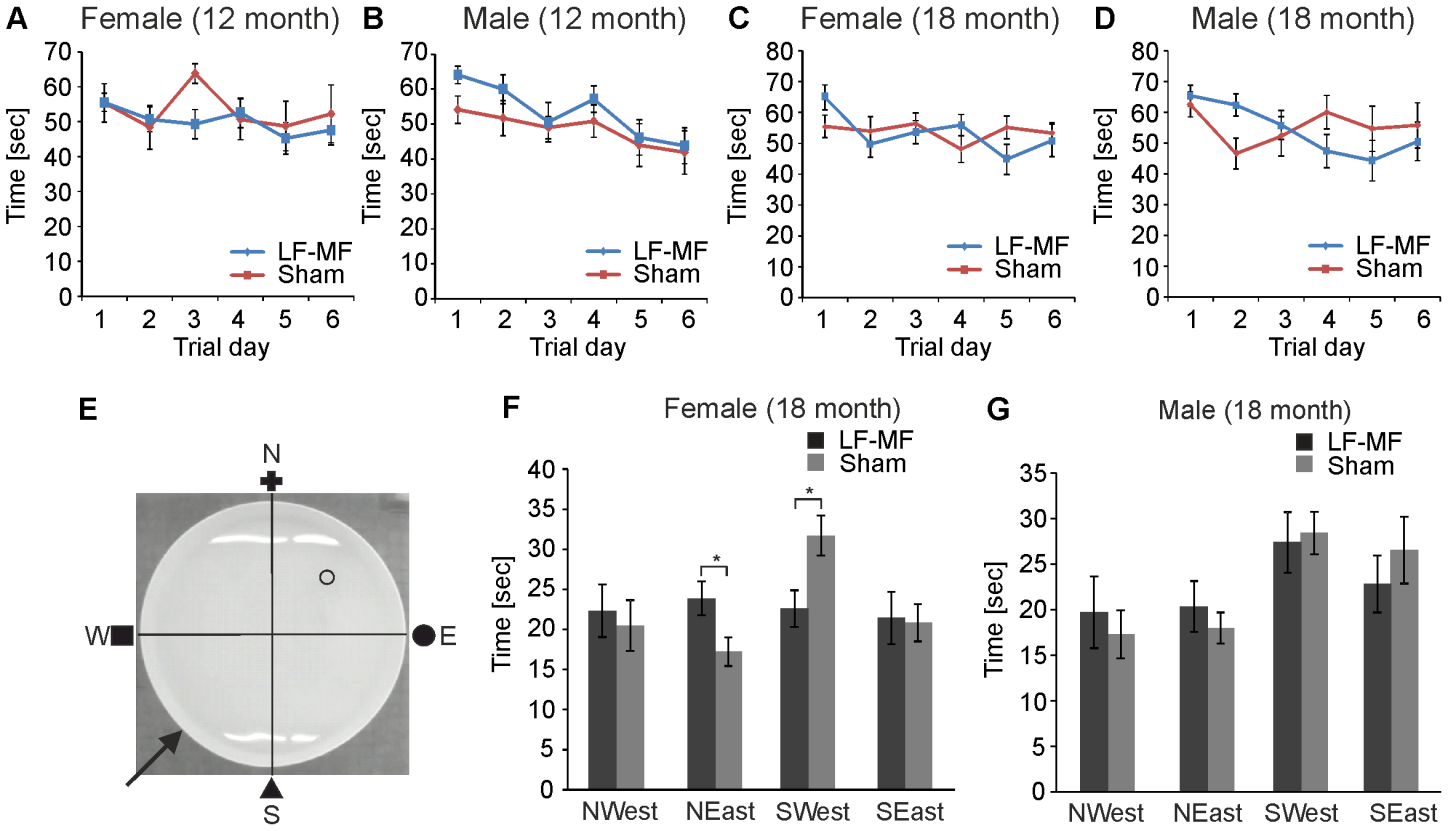
Spatial learning of APP23 mice upon exposure with LF-MF. Spatial learning of 12 month old (A, B) and 18 month old mice (C, D, F, G) was tested in a Morris water maze assay. (A–D) Mice were placed on six consecutive days in a tank surrounded by landmarks to find a hidden platform. The time mice needed to find the platform was monitored. (E–G) On day seven, a probe trial was performed whereby the platform was removed. All mice were placed in the south-west (SWest) quadrant of the pool (arrow in E). The time mice spent in each quadrant was monitored (A, B: male: LF-MF n = 8, sham n = 10; female: LF-MF n = 14, sham n = 6; C, D, F, G: male: LF-MF n = 8; sham n = 6; female: LF-MF = 9, sham n = 13; t-test; * p<0.05).

Our data indicate that the protein levels of APP as well as of soluble Aβ species were not altered in aged animals upon LF-MF exposure. To analyze whether APP-independent processes that might contribute to disease were affected by the exposure to LF-MF, we analyzed the ability of APP23 mice for spatial learning by employing a water maze assay. In this paradigm mice were trained to find a hidden platform after they were placed in a water tank that was surrounded by visual guidance cues (Fig. 3E). A learning curve was determined for six consecutive days with 12 month and 18 month old APP23 mice. No significant difference was detected after six days of learning in females and males of both ages (Fig. 3A–D). On the seventh day a probe trial without the platform was performed and the time that mice spent in each quadrant was determined. LF-MF did not adversely affect the behavior of both, female and male mice at 12 months (Supplemental Fig. 3) or 18 months of age (Fig. 3F,G) in this assay. It is of note that exposed female mice spent more time in the north-east quadrant (NEast) where the platform had been positioned previously compared to sham exposed animals, indicating a rather beneficial effect of LF-MF exposure. In contrast, no significant difference was observed with male mice. We therefore conclude that the long-term exposure to LF-MF did not adversely affect molecular processes that result in neuronal damage in APP23 mice.

### SOD1 expression and dismutase activity are independent of LF-MF exposure

**Figure 4:**
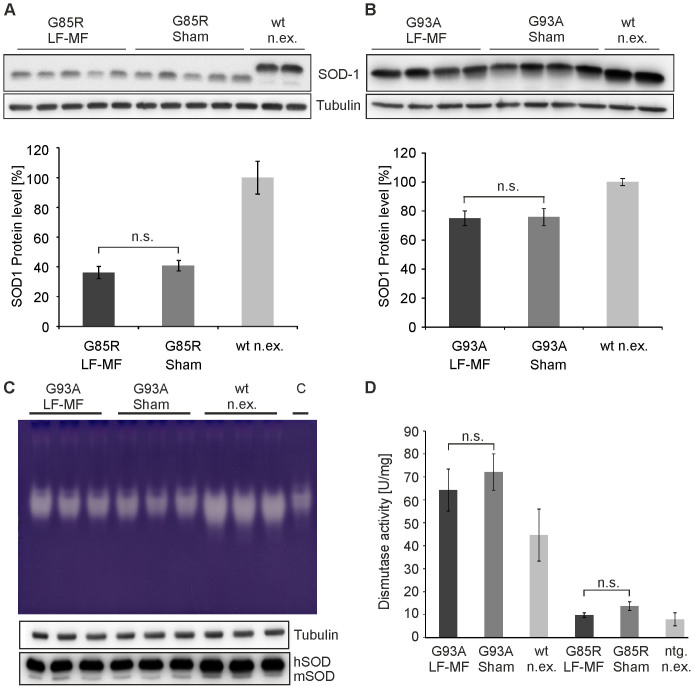
SOD1 protein levels and dismutase activity are unchanged upon exposure. (A, B) Total lysates from spinal cords of SOD^G85R^ (A), SOD1^G93A^ (B) and human SOD1^WT^ (wt n.ex.) mice were separated by SDS-PAGE and transferred on nitrocellulose membrane. SOD1 and tubulin were detected with specific antibodies by consecutive incubation on the same membrane (Supplemental Fig. 12). Bands of SOD1^G93A^ and human SOD1^WT^ run similar on the gels whereas SOD^G85R^ runs below human SOD1^WT^ and SOD1^G93A^. The densitometric analysis is shown in the corresponding lower panels. Data were normalized to SOD1 levels of non-exposed mice overexpressing SOD1^WT^ (wt n.ex.) (mean +/−SEM, n = 5; t-test). (C) Extracts from spinal cords of mutant SOD1 mice at endstage of disease and non-exposed age-matched SOD1^WT^ mice (wt n.ex.) and purified human SOD1 (“C”) were separated on native PAGE gels. Dismutase activity was determined by zymography. The bright bands represent areas of dismutase activity. SOD1 and tubulin protein levels were controlled by standard Western blot analysis by consecutive incubation of the respective antibodies on the same membrane (lower panel). (D) Dismutase activity was quantitatively determined relative to the total protein amount by a WST-assay and compared to non-exposed age matched SOD1^WT^ mice and nontransgenic littermates (ntg. n.ex.) (mean +/−SEM; n = 4 for mutant mice; n = 3 for controls; t-test, n.s. not sigificant).

Mutant SOD1 transgenic mice develop a late onset, progressive, and fatal ALS-like disease. Mice overexpressing SOD1^G85R^ and SOD1^G93A^ were placed in the exposure modules after genotyping with about two months of age until they reached endstage of disease at about 370 days and 297 days of age, respectively. Therefore SOD1^G85R^ and SOD1^G93A^ were continuously exposed for ten month and eight months, respectively. As the disease course in SOD1 transgenic mice is dose-dependent with regard to SOD1 protein levels and recent results indicate that the dismutase activity is enhanced upon LF-MF exposure^17^,^18^, expression levels and dismutase activity of SOD1 were determined. Total extracts from spinal cords of endstage animals were analyzed and no differences in protein levels (Fig. 4A, B) as well as in mRNA levels (Supplemental Fig. 4A, B) were observed in both transgenic mouse lines. In line with these data, the qualitative and quantitative analysis of dismutase activity was comparable between the LF-MF exposed and sham exposed cohorts (Fig. 4C,D; Supplemental Fig. 4C). It is of note that the dismutase activity levels in the liver of transgenic mice were also not altered upon exposure (Supplemental Fig. 4D), indicating that the results concerning SOD1 activity were not limited to the nervous system. In summary, LF-MF exposure has no effect on the expression and the activity of the disease-causing mutant SOD1 variants.

### Protein aggregation is not aggravated upon LF-MF exposure in SOD1 mice

**Figure 5:**
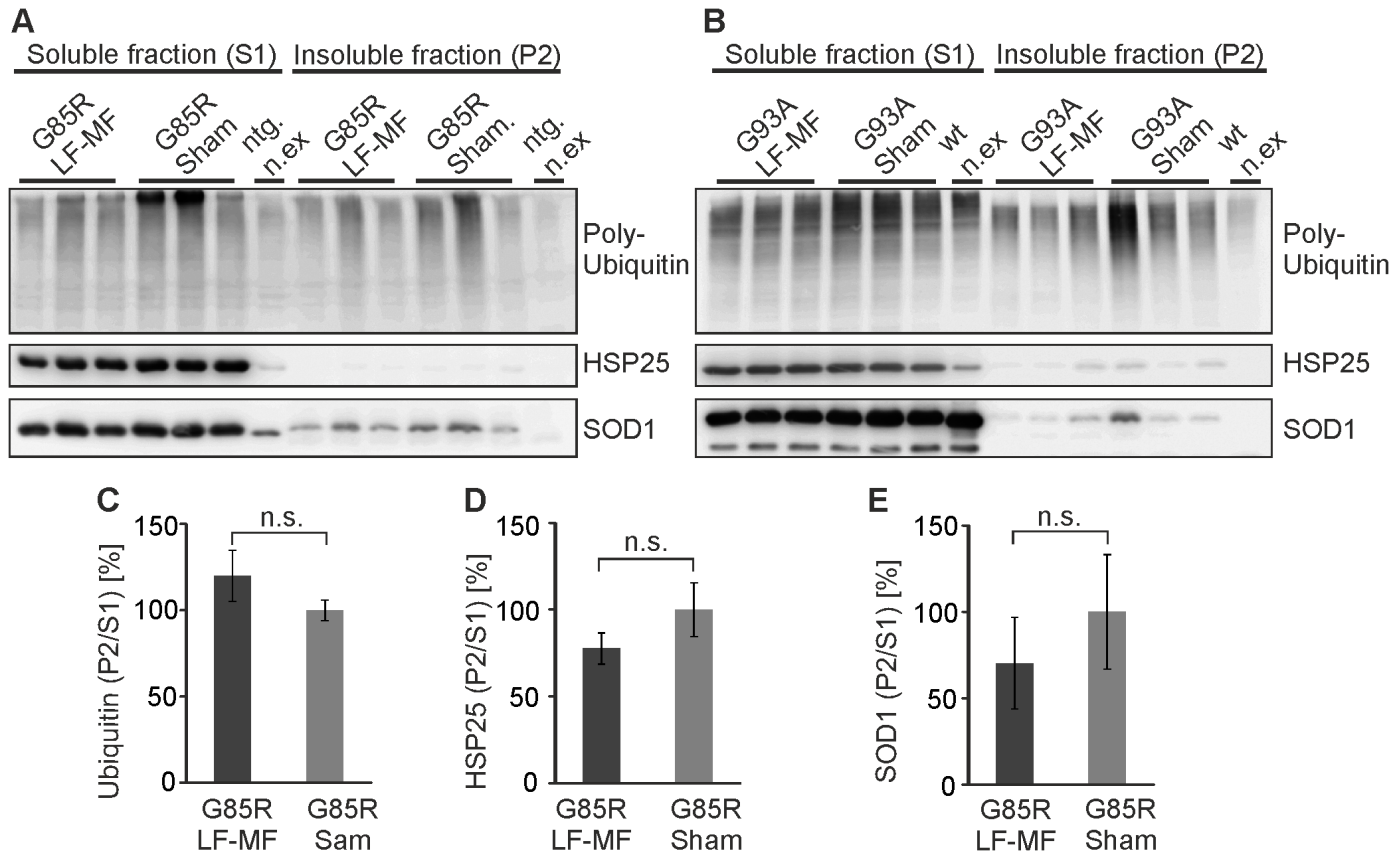
LF-MF exposure and protein aggregation. (A, B) Extracts from spinal cord tissues from female SOD1^G85R^ (A) and SOD1^G93A^ (B) mutant mice at endstage were separated by centrifugation. The soluble and the pellet fraction were analyzed by Western blot and polyubiquitinated proteins, HSP25 and SOD1 were detected by specific antibodies. Non-exposed non-transgenic littermates (ntg. n.ex.) and non-exposed SOD1^WT^ mice served as controls. The whole panels of the blots are displayed in Supplemental Fig. 13 and Supplemental Fig. 14 for SOD1^G85R^ and SOD1^G93A^, respectively. (C–E) For the densitometric analysis of Western blots the results from female and male SOD1^G85R^ mice (see Supplemental Fig. 5) were pooled. Results from exposed animals were normalized to sham exposed animals (mean +/−SEM; n = 6; t-test, n.s. not significant).

One pathological hallmark of ALS is the presence of protein aggregates in the affected tissues. Although it is still controversially discussed whether these aggregates containing SOD1 are causative for the disease, the formation of aggregates is reminiscent of a disturbed protein homeostasis. It has been proposed that LF-MF exposure alters protein degradation pathways^19^ and the gene expression as well as the protein levels of components relevant for proteostasis such as chaperones^20^. Here we analyzed whether the exposure to LF-MF alters the disease-associated accumulation of polyubiquinated proteins, SOD1, and HSP25 in detergent-insoluble fractions. Quantitative analysis of protein levels in spinal cords from SOD1^G85R^ and SOD1^G93A^ transgenic mice at endstage showed that the enrichment of these proteins in aggregates was not altered upon LF-MF exposure (Fig. 5, Supplemental Fig. 5). These data indicate that the protein network maintaining proteostasis was not sufficiently altered upon exposure to LF-MF to induce additional aggregation of misfolded or damaged proteins in diseased animals.

### Markers of oxidative stress are not elevated upon LF-MF exposure in mutant SOD1 mice

**Figure 6:**
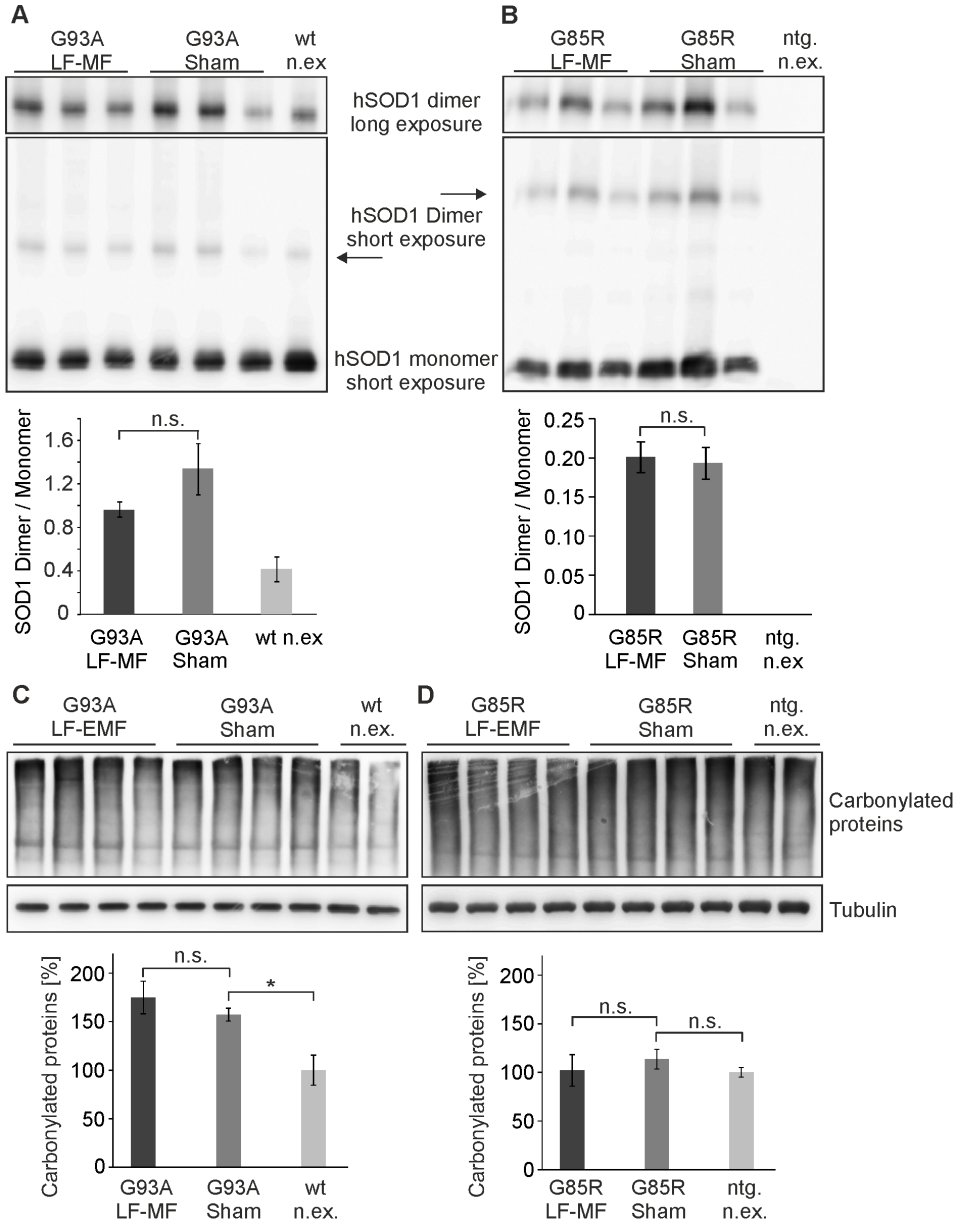
Oxidative markers are not modulated by LF-MF. (A, B) Total extracts from spinal cords of SOD1^G93A^ (A) and SOD1^G85R^ mice (B) were separated on PAGE gels without β-mercaptoethanol in the loading buffer. After Western blotting, SOD1 was detected with specific antibodies. Densitometric analysis is shown in lower panels. Data from SOD1^G93A^ and SOD1^G85R^ mice were compared to non-exposed mice overexpressing human wild-type SOD1 (wt n.ex.) or non-transgenic littermates (ntg. n.ex.), respectively. (mean +/-SEM; n = 6; t-test, n.s. not significant). (C, D) Carbonylated proteins in spinal cord extracts from female SOD1^G93A^ and SOD1^G85R^ mice at endstage were derivatized by dinitrophenylhydrazin (DNP) and detected by Western blot with specific antibodies directed against DNP. Tubulin served as a loading control and was developed on the same membrane. Results from SOD1^G93A^ and SOD1^G85R^ mice were normalized to non-exposed mice overexpressing human wild-type SOD1 (wt n.ex.) or non-transgenic littermates (ntg. n.ex.), respectively (mean +/−SEM, n = 8, t-test; * p<0.05).

There has been some evidence that the exposure to LF-MF has a pro-oxidant effect on different brain regions in mice and rats^9^. It is tempting to propose that this effect might aggravate the development of neurodegenerative diseases as the generation of reactive oxygen species (ROS) as a primary cause or secondary effect is a central aspect in the pathogenesis of ALS^21^,^22^. In order to analyze the oxidative status of the spinal cord at the endstage of disease, the amount of disulfate-linked SOD1 was determined in extracts from spinal cords after separation on non-reducing gels (Fig. 6A, B). The quantitative analysis revealed that the levels of covalently linked SOD1 dimers were not altered upon exposure. A more general measure for the oxidative burden of a tissue is the amount of carbonylated proteins that result from the presence of ROS by direct or indirect protein oxidation. In line with the previous results, protein carbonylation levels were not altered in endstage animals (Fig. 6C, D) indicating that the postulated pro-oxidative effect of LF-MF did not contribute to the increased oxidative burden in diseased mutant SOD1 animals.

### Glial activation is not increased upon LF-MF exposure in SOD1 transgenic mice

**Figure 7:**
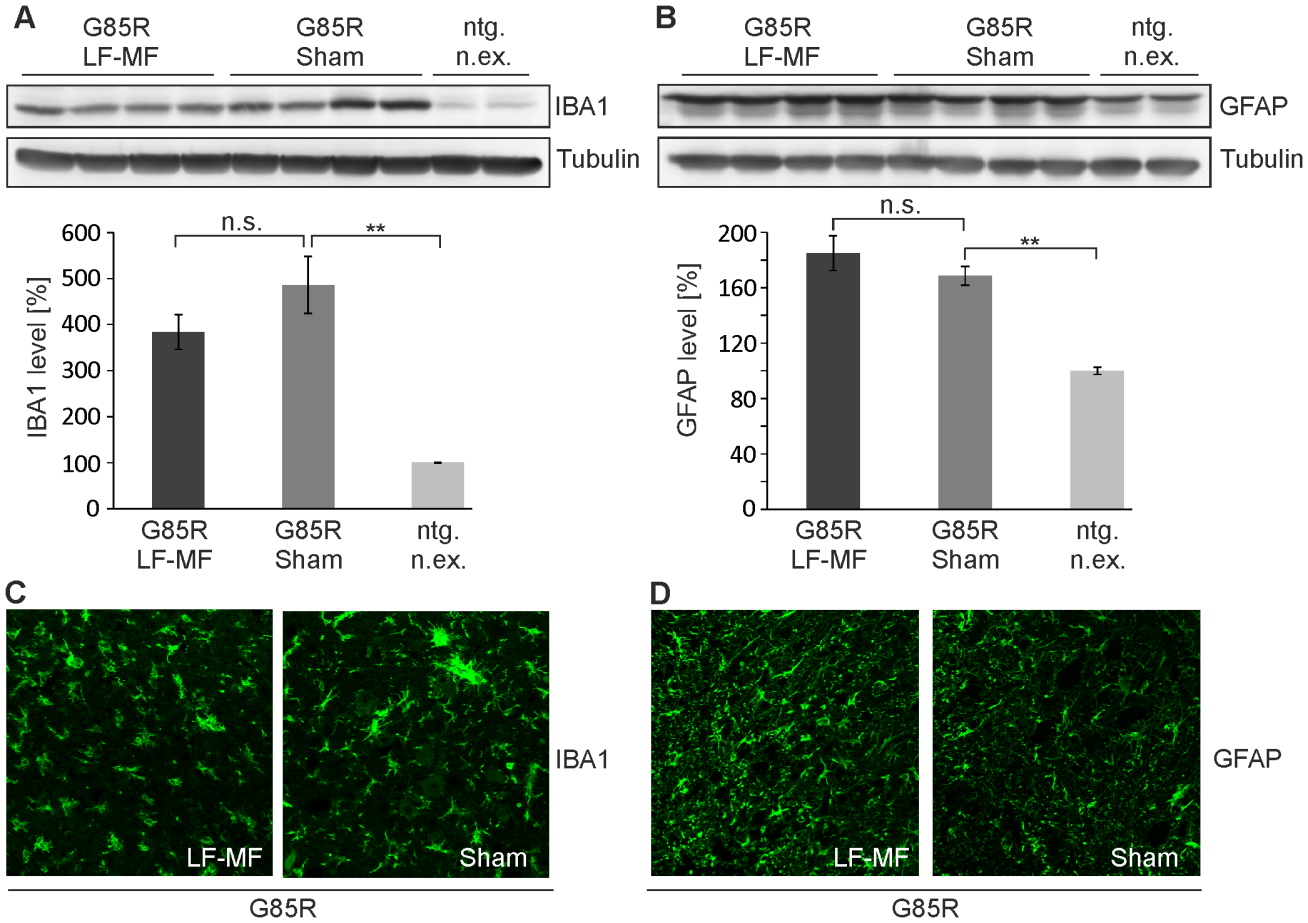
Microglia and astrocyte activation and LF-MF exposure. (A, B) Total lysates from spinal cords of SOD1^G85R^ mice were analyzed by Western blot. IBA1 (A) and GFAP (B) were detected with specific antibodies. The densitometric analysis is shown in corresponding lower panels. Tubulin served as a loading control and was developed on the corresponding membranes. Whole panels of the Western blots are displayed in Supplemental Fig. 17. Data were normalized to non-exposed littermates (ntg. n.ex.) (mean +/− SEM; n = 8, t-test; ** p<0.01). (C, D) Immunofluorescent staining with IBA1 (C) and GFAP antibodies (D) of spinal cord cross sections from endstage SOD1^G85R^ mice. Primary antibodies were detected with Cy2-conjugated species-specific secondary antibodies.

There is convincing evidence that microglia and astrocytes that are activated in the course of disease contribute to disease progression in SOD1 mouse models of ALS^23^,^24^. Although it is not entirely understood how glia cells contribute to disease on the molecular level, transplantation of glial-restricted precursor cells derived from mutant SOD1 transgenic mice induced pathology in wild-type animals^25^. Here we investigated whether the exposure to LF-MF had an effect on the levels of IBA1 and GFAP which are up-regulated upon microglia and astrocyte activation, respectively. IBA1 and GFAP protein levels in SOD1^G85R^ (Fig. 7A,B) and SOD1^G93A^ (Supplemental Fig. 6A,B) mutant lines were significantly increased compared to control animals, but no exposure-dependent difference was observed within mutant lines. The qualitative determination of IBA1 and GFAP in spinal cord cross sections confirmed the biochemical analysis (Fig. 7C,D; Supplemental Fig. 6C,D). These data indicate that the exposure to LF-MF did not affect the activation of glial cells under disease conditions.

### Disease course in mutant SOD1 mice is not altered upon exposure to LF-MF

**Figure 8:**
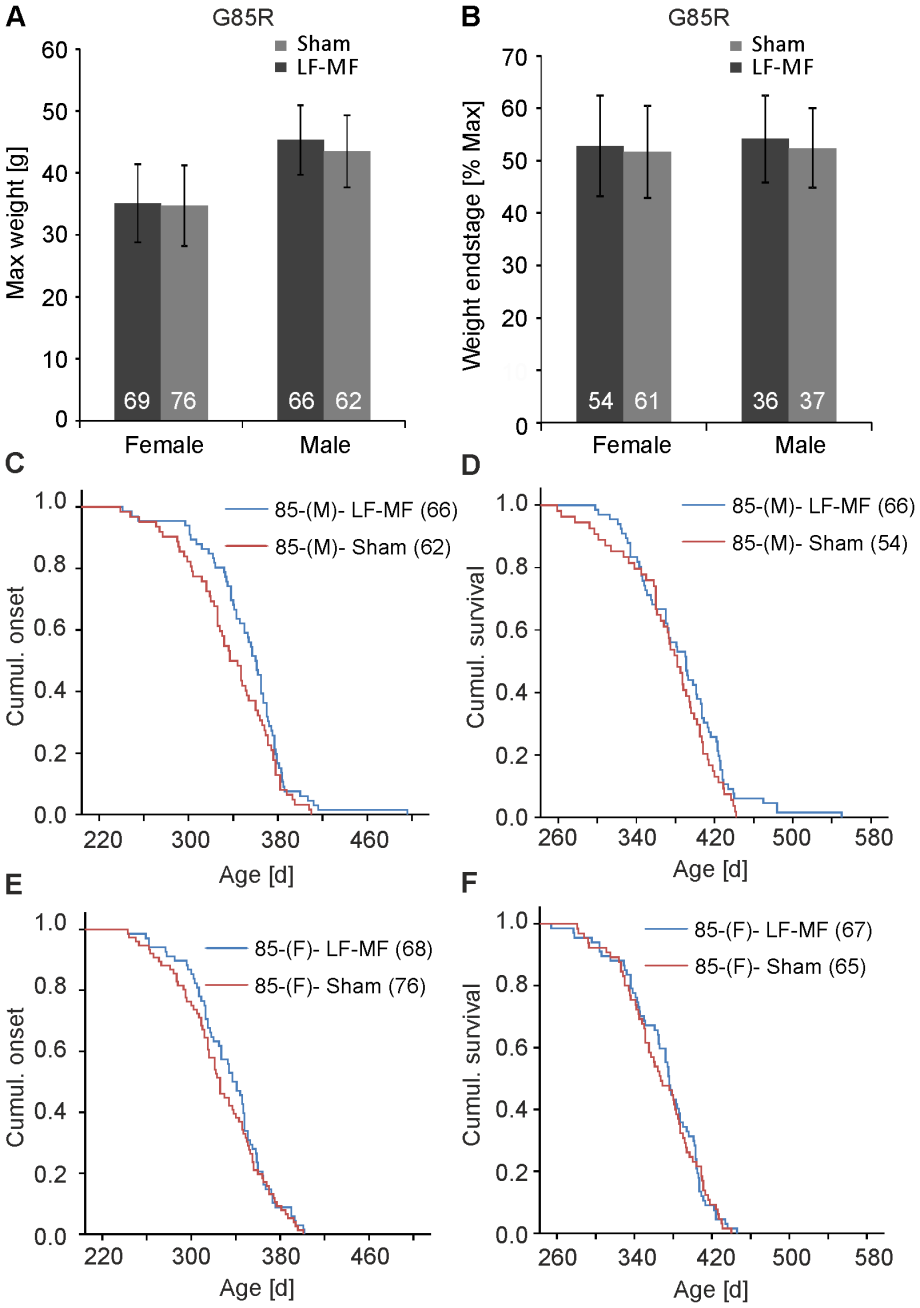
Disease onset and survival of SOD1^G85R^ mice are not affected by the exposure to LF-MF. The weight of mice was determined once a week. Age of onset was defined when mice lost 10% of their maximal weight. (A) Maximal weights of female and male mice under LF-MF or sham exposure conditions were determined and were not statistically altered upon exposure. The numbers within the bars represent the number of mice analyzed (mean +/− SEM; t-test). (B) Weights at endstage of disease are represented relative to the maximum weight per gender and exposure. (C, E) Kaplan-Meier curves show the probability of transgenic mice to be unaffected of disease at a given age. The onset of disease of male (C) and female (E) SOD1^G85R^ transgenic mice was not altered. The number in brackets shows the size of the cohort per condition analyzed. (D, F) Kaplan-Meier plot for the survival of female (F) and male (D) SOD1^G85R^ mice (Log-Rank-Test: C: p = 0.078; D: p = 0.184; E: p = 0.374; F: p = 0.782).

We have demonstrated that exposure to LF-MF has no adverse effect on processes that are proposed to be involved in ALS-pathogenesis like alterations of the proteostasis, the oxidative status or glial activation. As there has been no definite proof established that these processes are involved in disease induction, we analyzed the phenotypic disease progression in SOD1^G85R^ and SOD1^G93A^ mice, independent of any molecular mechanism. One measure for the vitality of the animals is the gain of weight in the pre-symptomatic phase. We did not observe an exposure-dependent alteration of the maximum weight gained in exposed male and female SOD1^G85R^ and SOD1^G93A^ animals (Fig. 8A; Supplemental Fig. 7A). Also the time of onset, which was defined when mice lost 10% of their maximum weight, was unaffected under exposure conditions in both mouse lines (Fig. 8C,E; Supplemental Fig. 7C,E). Exposed and sham exposed female mutant SOD1^G85R^ mice reached endstage of disease at a mean age of 370 d (+/− 5 d) and 367 (+/− 5 d), respectively (Fig. 8F). Also male SOD1^G85R^ mice (Fig. 8D; Supplemental Fig. 7B) and SOD1^G93A^ animals (Supplemental Fig. 7D,F) reached endstage of disease independently of exposure conditions. The observation that the weight at endstage was very similar between treatment groups (Fig. 8B) suggests that the disease course from onset to endstage of disease is unaffected upon exposure. These data indicate that the exposure to LF-MF neither affected pathways that initiate SOD1-mediated disease nor cellular pathways that result in the propagation of SOD1-mediated disease.

## Discussion

The exposure to LF-MF is a possible health risk as stated by the WHO^6^ and meta-analysis of epidemiological studies indicate that an increased occupational exposure might be a risk factor for the development of ALS and AD^3^,^4^. It is of note that in some studies the risk to develop AD upon the residential exposure to LF-MF was correlated with the strength of the magnetic field and the exposure time^26^. We have therefore conducted a large-scale animal study to investigate whether the continuous exposure to LF-MF (1 mT, 50 Hz) interferes with the pathogenesis of AD and ALS in suitable animal models. APP23 and the late-onset SOD1^G85R^ and SOD1^G93A^ mouse lines are well established and intensively characterized disease models. The mouse lines were chosen because they develop pathology in aged animals, allowing a LF-MF exposure for many months. APP23 mice were analyzed at 18 months of age after a continuous exposure for at least 16 months. In contrast to widely used transgenic mice that express multiple mutant AD-genes and develop an early onset of disease^27^, APP23 mice overexpress the Swedish APP double mutation which does not affect the survival rates and leads to the development of pathological hallmarks and learning deficits with increasing age^28^,^29^.

Transgenic mice overexpressing the SOD1^G85R^ and the SOD1^G93A^ mutant variants developed disease at 338 +/− 40 days and 263 +/− 28 days of age, respectively. SOD1^G85R^ and SOD1^G93A^ represent two classes of SOD1 mutations. The SOD1^G93A^ mutation shares many biophysical and biochemical properties of wild-type SOD1 despite it is structurally different to SOD1^WT^ 30,31. In contrast, dismutase activity of SOD1^G85R^ in tissues is not detectable^32^,^33^. One would predict that any interference of LF-MF with cellular pathways responsible for the disease would affect both mutant SOD1 lines. In summary, APP23 and mutant SOD1 lines are mouse models for AD and ALS that develop disease in aged animals allowing their experimental exposure to LF-MF for many months before disease onset. We therefore argue that this experimental setup is suitable for investigating the potential effect of LF-MF on the pathogenesis of AD and ALS and to investigate how LF-MF might affect relevant pathways on the cellular, the organ or the system level. However, based on the behavioral analysis of APP23 mice and the phenotypic progression of disease of transgenic SOD1 mice, no detrimental effect of LF-MF exposure was detected. A detailed biochemical analysis revealed that levels of disease-causing proteins, protein aggregation, oxidative damage and glial activation which are implicated to be important for the initiation and progression of disease were not altered upon LF-MF exposure.

Whether an increased exposure to LF-MF is a risk factor for ALS is controversially discussed. Epidemiological studies and subsequent meta-analysis reported inconsistent results^4^,^34^,^35^. We now conducted the first large cohort animal study that analyzed the impact of a long-term continuous exposure with LF-MF under defined laboratory conditions on the progression of ALS. This study significantly extends previous experiments^36^ for several reasons: (i) In the previous study by Poulltier de Gannes and colleagues (2009) a SOD1^G93A^ mouse line with a high copy number of the transgene was used which resulted in a medium survival time of about 140 days. The survival times of the mouse lines used in experiments presented here were about 297 days and 370 days for SOD1^G93A^ and SOD1^G85R^ mice, respectively. The longer mean survival time of mice used in our study correlates with lower levels and toxicity of mutant proteins which implies a higher susceptibility to extrinsic factors. (ii) As a consequence of the short life span of high-copy number SOD1^G93A^ mice, exposure in the previous study was applied for seven weeks. Moreover, mice were exposed only for two hours per day on five days per week^36^. In our study LF-MF were continuously applied for about eight and ten months for SOD1^G93A^ and SOD1^G85R^ mice, respectively. This is of particular interest, as the exposure time might be one of the critical determinants for LF-MF toxicity. Even under these conditions, exposure to LF-MF did not alter the age of disease onset and the survival of male and female mice of both mutant SOD1 mouse lines.

Although there is considerable evidence that the occupational and residential exposure to LF-MF might be a risk factor for AD^3^,^35^, so far no comprehensive study under controlled laboratory conditions had been conducted with relevant mouse models. Our results with APP23 mice now indicate that the exposure to LF-MF had no adverse effect on the learning behavior under disease conditions indicating that LF-MF do not interfere with the affected pathological pathways. It is of note that in this study exposed 18 month old female mice showed an improved learning behavior. This is in line with water maze experiments performed with normal male rats upon repeated exposure with LF-MF (4 weeks, 4 h/d, 2 mT, 50 Hz)^37^. The beneficial effect on learning behavior might be explained by evidence from electrophysiological studies *in vivo* or from acute hippocampal slices from exposed non-transgenic mice and rats, showing that LF-MF exerts a positive effect on synaptic activity and on long-term potentiation^38^,^39^,^40^. Despite this evidence points to a beneficial effect, some studies indicate that the exposure to LF-MF (25 d, 1 h/d, 1 mT, 50 Hz) might have adverse effects on the learning behavior of male mice in a Y-maze assay^41^. Nevertheless, one has to consider that the experimental paradigms (animal species, age, and gender; repeated vs chronic exposure; exposure time; magnetic field; learning test) were different in these studies.

In order to gain some mechanistic insights on how the exposure to LF-MF might interfere with pathogenesis in ALS and AD, we analyzed the levels of toxic protein species (mutant SOD1, APP, Aβ species), the appearance of protein aggregates which is reminiscent for an affected protein homeostasis (in mutant SOD1 spinal cords) and glial activation in affected tissues of APP23 and mutant SOD1 mice. In addition, we determined the oxidative damage in mutant SOD1 mice as it has been proposed by a variety of studies with different cellular and animal systems that LF-MF might exerted a pro-oxidant effect in the nervous system^9^. Our data indicate that LF-MF did not alter the aforementioned pathogenic pathways on the molecular level in both disease model systems.

In summary, our study suggests that a continuous exposure of large cohorts of transgenic ALS and AD mouse models between 10 and 18 months does not aggravate cellular processes involved in the progression of both diseases. In line with these results, behavioral and disease course assessments were not altered upon exposure. These unbiased readouts indicate that exposure to LF-MF does not affect as yet unknown pathological processes that might be involved in the induction of AD and ALS pathogenesis.

## Methods

### Animals

Mice were kept in the animal facility of the University Medical Center and housed, treated, anesthetized, and sacrificed according to the state law of Rheinland-Pfalz and German and European guidelines for the care and use of laboratory animals. All animal procedures were approved by the state of Rheinland-Pfalz. Animals were kept under a 12 h light-dark cycle with food and drinking water *ad libitum*. APP23^28^ mice expressing the Swedish double mutation of APP and SOD1^G85R^ 13 and SOD1^G93A^ mice^14^ were exposed to LF-MF or sham exposed after genotyping. The weight of all mice was determined once a week. Onset of disease of transgenic SOD1 mice was defined as the time point when mice lost 10% of their maximum weight. The endstage of disease was determined when animals were not able to right themselves up within 10 sec when put on the side. Body temperature of mutant SOD1 mice was monitored by a subcutaneously implanted transponder (BioMedic Data Systems) once a week, always at the same time. The body temperature of exposed and sham exposed animals was comparable (Supplemental Fig. 8).

### Exposure unit

**Figure 9:**
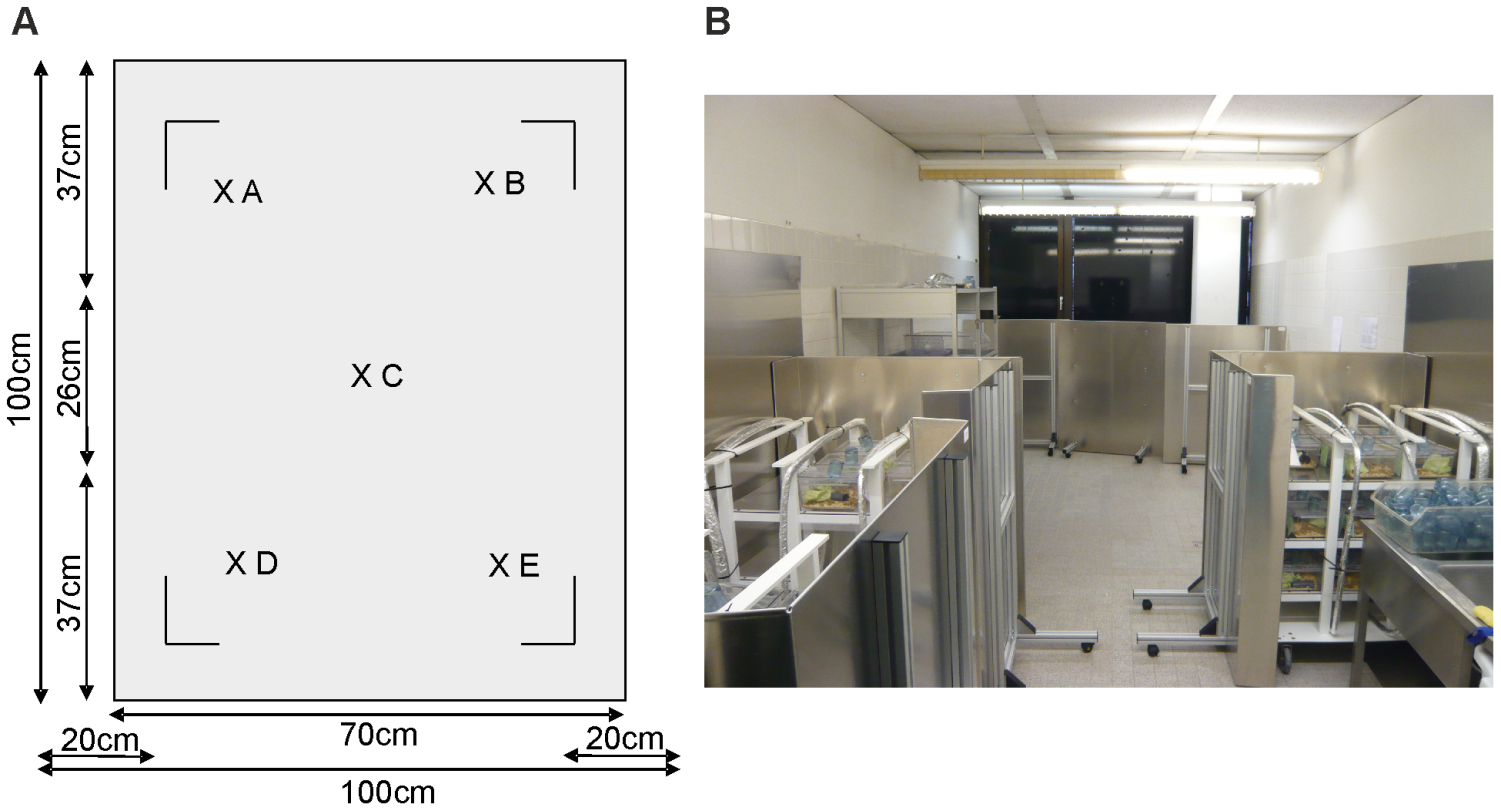
Exposure unit. (A, B) Animals were housed in cages without any metal content and placed within Merrit coils. The electric field was shielded by wrapping the wires in aluminum foil. Exposure modules were designed such that cages could be placed on three levels. (A) The homogeneity of the field on each platform was controlled regularly at five spots as indicated on all three levels of the module. (B) The exposure units were shielded to prevent fringing.

Animals were placed in Merritt coils^42^ that generate a homogenous magnetic field and allow the exposure of several mouse cages in one module (Fig. 9B). Merritt coils consisted of two coils of 26 and 11 turns each which were connected in series and generated a magnetic field of 1 mT (Fig. 9A). The cable consisted of five wires with a total cross-section area of 16 mm^2^. Modules for sham exposure looked identical, but wires were turned after half of the turns. The magnetic field in the latter modules was at largest 2 µT. Wires were wrapped in aluminum foil to block electric fields. Alternating currents (50 Hz) were generated by the IP20 power supply from Bürger Elektronik. Magnetic flux density was monitored regularly (EMF-822 Teslameter, Lutron Electronic Enterprise; Koshava 4, Wuntronic) and the magnetic field was shielded by PowerShield (Systron) to avoid fringing. Cages and water bottles were without metal content.

### Morris water maze

To test spatial learning and memory, a Morris water maze hidden platform task was performed^29^,^43^,^44^. Mice were placed in a circular water tank (1.5 m in diameter) surrounded by prominent objects that provide spatial guidance cues. The task was to find a hidden platform (15 cm in diameter) located 1.5 cm below the water surface. To accustom mice to the system, animals were placed in the water with a visible platform in the northeastern quadrant for 3 min. Training trials with the hidden platform were performed on six consecutive days where mice were placed in the tank four times in at different positions on one given day. Mice were guided to the platform after 70 sec when the platform was not found and all mice were allowed to stay on the platform for 30 sec. For the probe trial on day seven, all mice were placed in the southwest quadrant and the time mice spent in each quadrant was determined. All trials were monitored and analyzed with the EthoVision System (Noldus).

### Quantitative real-time PCR

RNA was prepared from spinal cords of endstage SOD1 transgenic mice with the RNeasy Lipid Tissue Mini Kit (Qiagen) according the manufacturer's instructions. To generate cDNA from purified RNA, Superscript III First-Strand Synthesis SuperMix for qRT-PCR (Life technologies) was used. Real-time PCR was performed with the iCycler (BioRad) using the appropriate primers (Supplemental Table 1) and the SYBR-green Sensimix (Bioline). Housekeeping genes beta-actin and L19 served as a control.

### Tissue extraction and aggregate enrichment

To generate total lysates, tissues were homogenized with a glass homogenizer and sonicated in Tris-HCL (pH 6.8) supplemented with 10% (w/v) sucrose, 2% (w/v) SDS and a proteinase inhibitor cocktail with EDTA (Roche). Detergent-resistant aggregate-enriched fractions from spinal cord were generated by homogenizing and sonication of tissues in a hypotonic buffer (HEPES 10 mM, 10 mM NaCl, 5 mM NaHCO_3_, 5 mM EDTA, 1 mM KH_2_PO_4_, 1 mM CaCl_2_, 0.2 mM MgCl_2_, pH 7.4). Total lysates were separated in a soluble and a pellet fraction by centrifugation (6500 g, 4°C). The soluble fraction was carefully removed for analysis and the pellet was washed for two times under the same conditions. After the final wash, the pellet was resuspended in TSE-buffer containing 10 mM Tris-base, 200 mM sucrose, 1 mM EDTA, 1% TritonX-100, 1% Nonidet, pH 7.5. The lysate was separated in a soluble and a pellet fraction by centrifugation (4000 *g*, 4°C) and the pellet fraction was washed. The final pellet was resuspended in TSE buffer and used for analysis. All buffers were supplemented with a proteinase inhibitor cocktail (Roche). Protein concentrations of the different fractions were determined by a BCA-assay (Pierce).

### SDS-PAGE and Western blot

Equal amounts of proteins were supplemented with SDS- and ß-mercaptoethanol-containing loading buffer and separated on SDS-PAGE gels as indicated. Proteins were transferred on nitrocellulose membranes and subsequently detected by specific antibodies to APP (6E10, Covance), DNP (Molecular Probes), GFAP (Dako), HSP25 (Enzo), IBA1 (Wako), SOD1 (Epitomics), Tubulin (DM1A, Sigma), and Ubiquitin (Dako). Primary antibodies were recognized by species-specific secondary antibodies coupled to horseradish-peroxidase and developed by enhanced luminescence. Signals were detected by the Fusion system (Peqlab) or the LAS Image Analyzer (Fuji) and analyzed using Image J software.

### Assessment of dismutase activity

A semi-quantitative determination of dismutase activity was performed by zymography as described earlier^33^. In brief, total lysates were generated by homogenization of tissues in hypotonic buffer (see *Tissue extraction and aggregate enrichment*). Equal protein amounts were separated on native gels without SDS and gels were stained for 45 min in the dark with 50 mM KPO_4_ (pH 7.4) containing 275 µg/ml NBT, 65 µg/ml riboflavin and 3,2 µl/ml TEMED and scanned for documentation. To quantitatively determine dismutase activity in tissue extracts, spinal cords were homogenized in hypotonic buffer (see above) and separated in a soluble and a pellet fraction by centrifugation (100,000 g, 4°C, 30 min). Dismutase activity of the soluble fraction was determined with the WST Assay kit according to the manufacturer's protocol (Dojindo Molecular Technologies).

### Quantitative determination of Aβ levels by ELISA

Tissue lysates from cortex and hippocampus of APP23 mice were prepared in PBS. After a centrifugation step (16,000 g, 20 min), supernatants were kept and 1 µg of total protein was used for the determination of Aβ_(40)_ and Aβ_(42)_ levels according to the manufacturer's protocol (Life Technologies).

### Immunofluorescent staining

Transgenic mice at endstage of disease were deeply anesthetized and perfused with PBS and subsequently with 4% paraformaldehyde. Spinal cord tissues were removed, incubated in 30% (w/v) sucrose and 15 µm thick frozen sections were generated (CM1900, Leica). Sections were blocked in 10% serum and primary IBA1 (Wako) and GFAP (Dako) antibodies or tomatolectin (Sigma) were incubated over night at 4°C. Fluorescently labelled secondary antibodies were incubated for 1 h at room temperature. Stainings were documented with a confocal microscope (LSM710, Zeiss).

### Kongo red staining

One hemisphere of brains from APP23 mice was fixed in 4% paraformaldehyde and embedded in embedding medium (Jung). About 200 frozen sections (10 µm) were prepared and categorized upon anatomical landmarks in frontal, medial and caudal sections. Sections were stained with hematoxilin and subsequently with 0.5% (w/v) kongo red. Washed and dried sections were documented with a Zeiss Axiovert microscope equipped with a Spot Insight QE (histological pictures) or a Spot-RT camera (fluorescent pictures; Visitron). Pictures were processed with ImageJ to increase the contrast and the number of plaques per brain region was counted. Data were confirmed by an independent assessment of blinded sections.

### Statistics

Statistical analysis was performed with SPSS software (IBM Software). The statistical test is specified with each figure.

## Author Contributions

M.P.L., J.W., A.S.B., A.-K.S. planned, performed and analyzed experiments and contributed to the writing of the manuscript; H.R. designed the exposure modules; C.B. and A.M.C. designed the study, analyzed the experiments and wrote the manuscript.

## Supplementary Material

Supplementary Information Liebl et al Supplementary Information
