# Supplementary material for: Low-frequency magnetic fields do not aggravate disease in mouse models of Alzheimer's disease and amyotrophic lateral sclerosis
Source: Sci Rep. 2015 Feb 26;5:8585. doi: 10.1038/srep08585 (PMC4341214; doi:10.1038/srep08585)
Supplement: Supplementary Information — Liebl et al Supplementary Information [file srep08585-s1.pdf]

Low-frequency magnetic fields do not aggravate disease in mouse models of Alzheimer's disease and amyotrophic lateral sclerosis

Martina P. Liebl[1], Johannes Windschmitt[1], Anna S. Besemer[1], Anne-Kathrin Schäfer[1], Helmut Reber[2], Christian Behl[1], Albrecht M. Clement[1]

[1] Institute for Pathobiochemistry and [2] Department of Nuclear Medicine, University Medical Center of the Johannes Gutenberg University, Mainz, Germany

Supplemental material

Supplemental Fig. 1: LF-MF exposure does not induce the generation of Aβ plaques in APP23 mice. (A, B) Kongo red staining of a medial brain section from a 18 month old female APP23 mouse. Signals overlap in light microscopic (A) and fluorescent pictures with 545 nm excitation (B). (C, D) Composite of fluorescent pictures of medial sections of exposed (C) and sham exposed (D) female mice with 18 months of age in which the brain stem and the diencephalon were removed. Areas representing the cortex and the hippocampus are highlighted (D). (E-G) Number of plaques in the cortex and the hippocampus in frontal (E), medial (F) and caudal (G) brain sections were counted after processing the composite pictures with ImageJ (mean +/-SEM; LF-MF n=3, sham n=4; t-test; n.s. not significant).

Supplemental Fig. 2: Analysis of sAPPα and sAPPβ in exposed and sham exposed APP23 mice. sAPPα (A, B) and sAPPβ (C, D) levels in total lysates of cortex (A, C) and hippocampus (B, D) of 18 month old female APP23 mice were determined by Western blot. sAPPα is represented by the lower band, the upper band represents full-length APP (A, B). Endogenous mouse APP cannot be detected under these conditions. Tubulin served as a loading control and was applied consecutively on the same corresponding membranes (Supplemental Fig. 10) (mean +/-SEM; sAPPα: cortex n=11 for LF-MF and sham, Mann-Whitney-U-Test; hippocampus LF-MF n=10, sham n=12, t-test; sAPPβ: cortex 10 for LF-MF and sham, Mann-Whitney-U-Test; hippocampus LF-MF n=10, sham n=13, t-test, n.s. not significant).

Supplemental Fig. 3: Spatial learning of 12 month old APP23 mice. The spatial learning behavior of male (A) and female (B) APP23 mice at 12 months of age was investigated by a Morris water maze test. After six consecutive days of training to find a hidden platform in a

water tank, a probe trial was performed in which the platform was removed. The time that mice spent in the relevant quadrants of the tank was monitored (mean +/-SEM; male: LF-MF n=8, sham n=10; female: LF-MF n= 14, sham n=6).

Supplemental Fig. 4: SOD1 mRNA levels in spinal cords and dismutase activity in tissues of transgenic SOD1 mice. (A, B) Expression levels of human SOD1 (hSOD1) (A) and mouse (mSOD1) (B) were determined in spinal cords of mutant SOD1 lines, SOD1^{WT} mice (wt) and non-transgenic littermates by real-time PCR. Expression levels were compared to the housekeeping genes actin and 60S ribosomal protein L19. hSOD1 levels were normalized to the expression in SOD1^{WT} mice (A) and mSOD1 levels were normalized to expression levels in non-transgenic littermates (B) (n=4, mean +/- SEM, t-test to compare exposed and sham exposed mice, all pairs not significant). (C) Extracts form the spinal cord of SOD1^{G85R} mice at endstage of disease, non-exposed age-matched SOD1^{WT} mice (wt n.ex.) and non-transgenic littermates (ntg. n.ex.) and purified human SOD1 ("C") were separated on native PAGE gels. Dismutase activity was determined by zymography. The bright bands represent areas of dismutase activity. Protein levels were controlled by standard Western blot analysis. Antibodies against SOD1 and tubulin were applied consecutively on the same membrane (lower panel). (D) Dismutase activity in liver extracts was quantitatively determined relative to the total protein amount by a WST-assay (mean +/-SEM; n=4 for mutant SOD1 mice; n=3 for SOD1^{WT} und non-transgenic mice, t-test).

Supplemental Fig 5: LF-MF exposure and protein aggregation in mutant SOD1 mice. (A, B) Extracts from spinal cord tissues from male SOD1^{G85R} (A) and SOD1^{G93A} mice (B) at endstage

were separated by centrifugation. The soluble and the pellet fractions were analyzed by Western blot and polyubiquitin, HSP25, and SOD1 were detected by specific antibodies. Non-exposed non-transgenic littermates (ntg. n.ex.) and $SOD1^{WT}$ mice (wt n.ex.) served as a control. Whole panels of the Western blots are displayed in Supplemental Fig. 15 and Supplemental Fig. 16 for $SOD1^{G85R}$ and $SOD1^{G93A}$ mice, respectively. (C –E) For the densitometric analysis of Western blots the results from female (see Fig. 5) and male $SOD1^{G93A}$ mice were pooled. Results from exposed animals were normalized to sham exposed animals. (mean +/-SEM; n=6; t-test, n.s. not significant)

Supplemental Fig 6: Microglia and astrocyte activation and LF-MF exposure in $SOD1^{G93A}$ mice. (A, B) Total lysates from spinal cords of $SOD1^{G93A}$ mice were analyzed by Western blot. IBA1 (A) and GFAP (B) were detected with specific antibodies. The densitometric analysis is shown in corresponding lower panels. Tubulin served as a loading control and was analyzed on the same corresponding membranes. Whole panels of the Western blots are displayed in Supplemental Fig. 17. Data were normalized to non-exposed $SOD1^{WT}$ mice (wt n.ex.) (mean +/- SEM; n=8, t-test). (C, D) Immunofluorescent stainings with IBA1 (C) and GFAP antibodies (D) of spinal cord cross sections from endstage $SOD1^{G93A}$ mice. Primary antibodies were detected with Cy2-conjugated species-specific secondary antibodies.

Supplemental Fig. 7: Disease onset and survival of $SOD1^{G93A}$ mice are not affected by the exposure to LF-MF. The weight of mice was determined once a week. The age of disease onset was determined when mice lost 10 % of their maximal weight. (A) Maximal weights of female and male mice under LF-MF or sham exposure conditions were determined and were not statistically altered upon exposure. The numbers within the bars represent the number

of mice analyzed (mean +/- SEM; t-test). (B) Weights at endstage of disease are represented relative to the maximum weight per gender and exposure. (C, E) The onset of disease of male (C) and female (E) $SOD1^{G93A}$ transgenic mice was not altered. Kaplan-Meier curves show the probability of transgenic mice to be unaffected by disease at a given age. The number in brackets shows the size of the cohort per condition analyzed. (D, F) Kaplan-Meier plot for the survival of male (D) and female (F) $SOD1^{G93A}$ mice (Log-Rank-Test: C: p=0.165; D: p=0.475; E: p=0.855; F: p=0.681).

Supplemental Fig. 8: Body temperature of SOD1 transgenic mice was not different between cohorts. In order to exclude that temperature might influence the outcome of the experiments, body temperature of five $SOD1^{G93A}$ mice under either condition was assessed after subcutaneous implantation of a transponder. The temperature of mice was monitored until the first mouse reached onset of disease. Except for one time point, temperatures were not significantly different between cohorts (mean +/-SEM, n=5 for LF-MF and sham, t-test,* $p \leq 0.05$).

Supplemental Fig. 9: Original Western blot panels corresponding to Fig. 1A and B. App antibody (6E10) was incubated and developed prior to the tubulin detection on the same membrane. Protein standards were pasted on the original blot. The boxes represent the bands shown in the original figure.

Supplemental Fig. 10: Original Western blot panels corresponding to Supplemental Fig. 2. sAPPα was detected with the 6E10 antibody (A, B). sAPPβ was detected with specific

antibodies (C, D). Tubulin was detected on the same corresponding membranes. The boxes represent the bands shown in the original figure.

Supplemental Fig. 11: Original Western blot panels corresponding to Fig. 2. IBA1 (A, B) and GFAP (C, D) were detected with specific antibodies. Tubulin was detected on the same corresponding membranes. The boxes represent the bands shown in the original figure.

Supplemental Fig. 12: Original Western blot panels corresponding to Fig. 4. SOD1 was detected with a specific antibody (rabbit monoclonal from Epitomics) that primarily recognizes human SOD1. The $SOD1^{G85R}$ variant runs below human $SOD1^{WT}$ but above endogenous mouse SOD1 (A). The $SOD1^{G93A}$ variant runs indistinguishable from human $SOD1^{WT}$ (B). Tubulin was developed after the detection of SOD1. The boxes represent the bands shown in the original figure.

Supplemental Fig. 13: Original Western blot panels corresponding to Fig. 5A. Extracts from spinal cord tissues from female $SOD1^{G85R}$ mice at endstage were fractionated in a soluble and an aggregate-enriched fraction and separated on SDS-PAGE. Polyubiquitin, SOD1, HSP25 were detected by Western Blot on the same membrane. All Western blots had been performed under the same experimental conditions. The boxes represent the bands shown in the original figure.

Supplemental Fig. 14: Original Western blot panels corresponding to Fig. 5B. A soluble and an aggregate-enriched fraction from spinal cord tissues from female $SOD1^{G93A}$ mice at endstage were separated on SDS-PAGE. Polyubiquitin, SOD1, HSP25 were detected by

Western Blot. Polyubiquitin and HSP25 were detected on the same membrane. All Western blots had been performed under the same experimental conditions. The boxes represent the bands shown in the original figure.

Supplemental Fig. 15: Original Western blot panels corresponding to Supplemental Fig. 5A. Extracts from spinal cord tissues from male $SOD1^{G85R}$ mice at endstage were separated by SDS-Page and polyubiquitin, SOD1, and HSP25 were detected after transfer on a nitrocellulose membrane. SOD1 and HSP25 were detected on the same membrane in consecutive incubations. Western blots had been performed under the same experimental conditions. The boxes represent the bands shown in the original figure.

Supplemental Fig. 16: Original Western blot panels corresponding to Supplemental Fig. 5B. Extracts from spinal cord tissues from male $SOD1^{G93A}$ mice at endstage were separated and the soluble and an aggregate-enriched fractions were analyzed by Western blot. Polyubiquitin, SOD1, HSP25 were detected on different membranes, but Western blots had been performed under the same experimental conditions. The boxes represent the bands shown in the original figure.

Supplemental Fig. 17: Original Western blot panels corresponding to Fig. 7 and Supplemental Fig. 6. GFAP (A, B) and IBA (C, D) were detected with specific antibodies. Tubulin was detected on the same corresponding membranes. The membranes for the detection of IBA1 were cut in half directly after the transfer. The boxes represent the bands shown in the original figure.

Supplemental Tables

Supplemental Table 1: Primer sequences for RT-qPCR. Expression levels of human and mouse SOD1 were determined and normalized to the expression of actin (Actg1) and 60S ribosomal protein L19 (RPL19).

**Gene**	**Orientation**	**Sequence**	**Species**
SOD1	Forward	5´-TGG CCG ATG TGT CTA TTG AA-3´	*Homo sapiens*
SOD1	Reverse	5´-TTA CAC CAC AAG CCA AAC GA-3´	*Homo sapiens*
SOD1	Forward	5´-AAC CAG TTG TGT TGT CAG GAC	*Mus musculus*
SOD1	Reverse	5´-CCA CCA TGT TTC TTA GAG TGA GG	*Mus musculus*
Actg1	Forward	5´-TGG ATC AGC AAC CAG GAG TAT G-3´	*Mus musculus*
Actg1	Reverse	5´-CCT GCT CAG TCC ATC TAG AAG CA-3´	*Mus musculus*
RPL19	Forward	5´-GAA ATC GCC AAT GCC AAC TC-3´	*Mus musculus*
RPL19	Reverse	5´-TTC CTT GGT CTT AGA CCT GCG-3´	*Mus musculus*

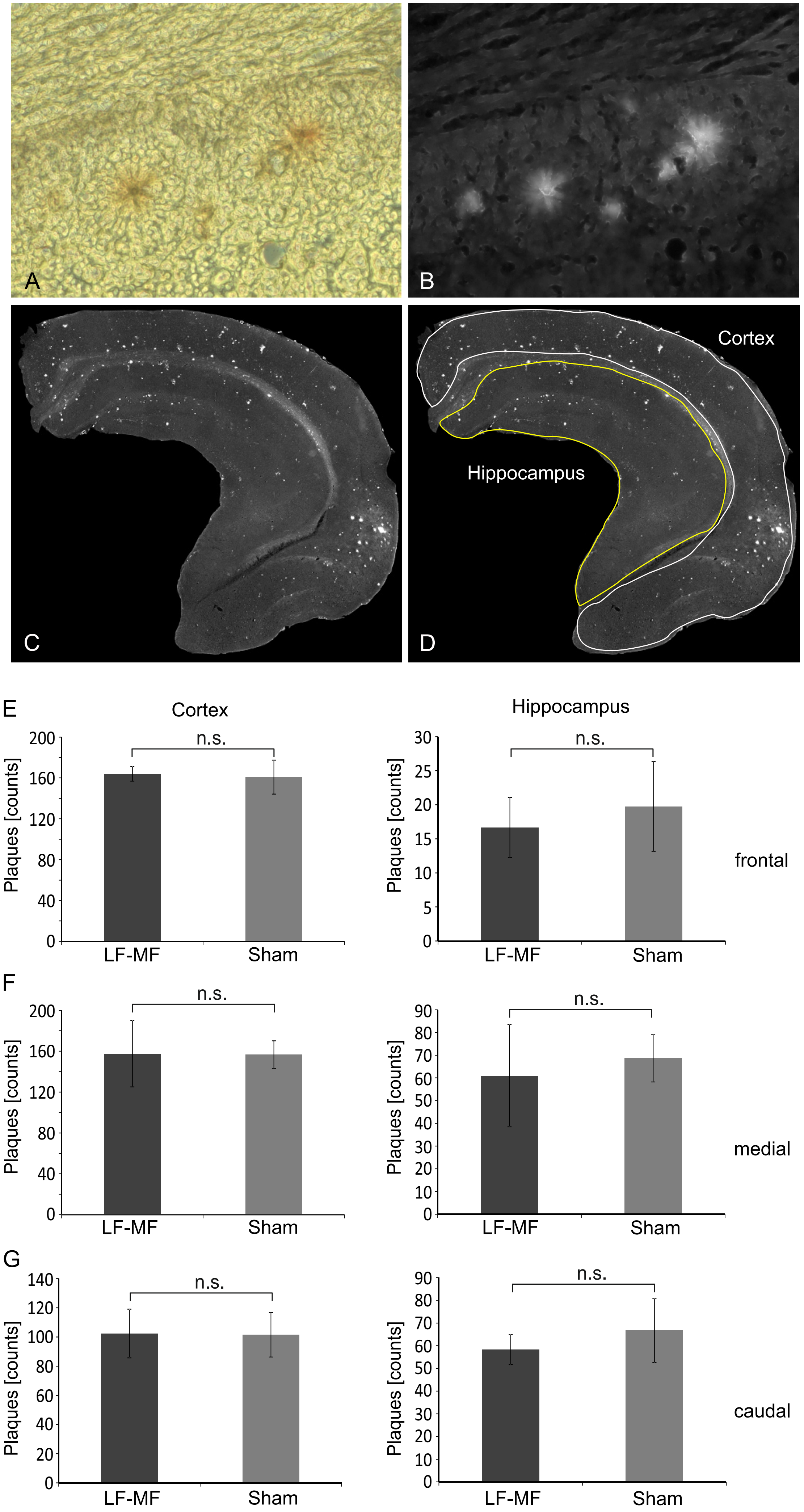
A
B
C
D
Cortex
Hippocampus
E
Cortex
Hippocampus
frontal
F
medial
G
caudal
Plaques [counts]
n.s.
LF-MF
Sham

Suppl Fig. 1

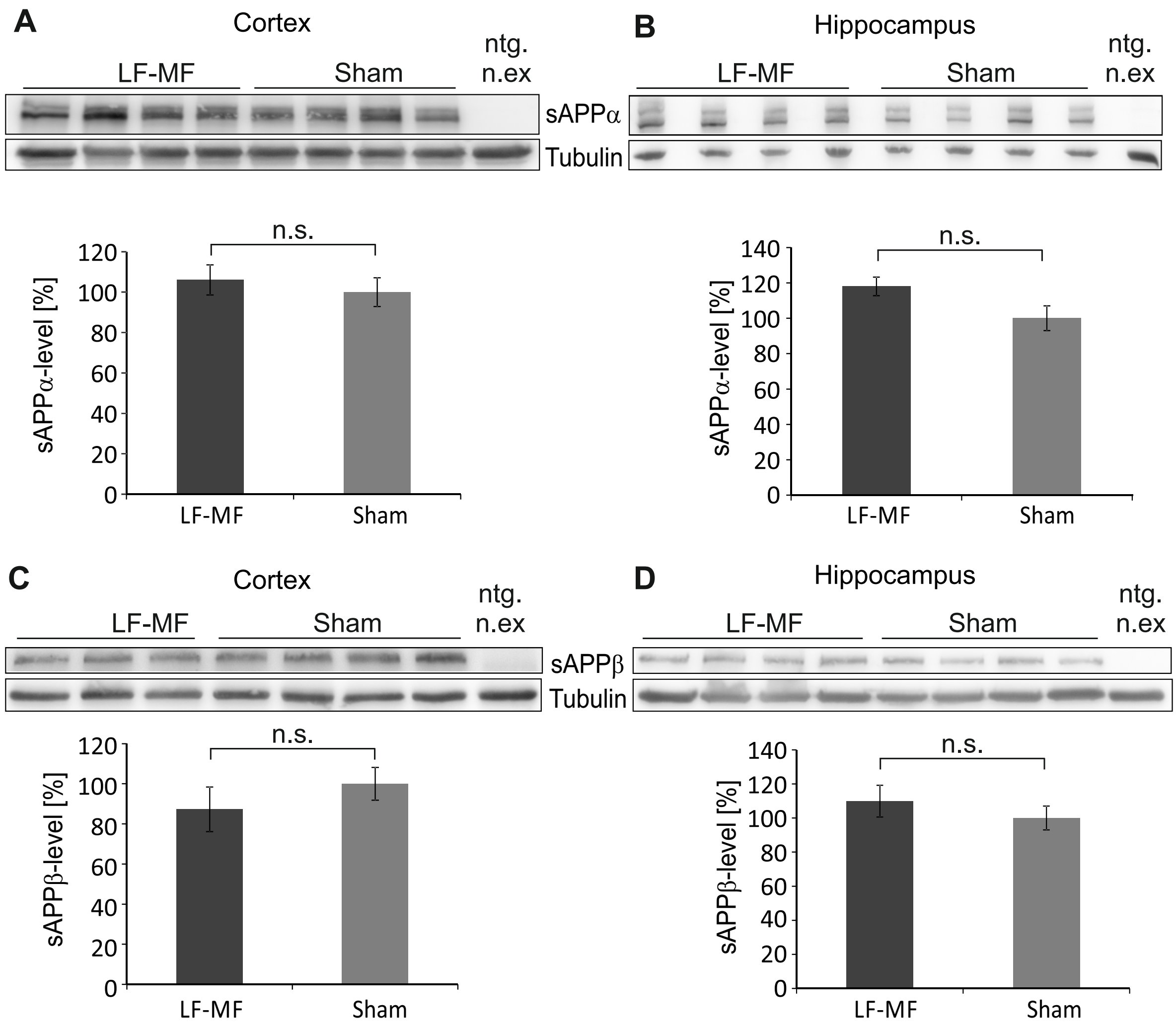
A
Cortex
LF-MF
Sham
ntg.
n.ex
sAPPα
Tubulin
n.s.
sAPPα-level [%]
LF-MF
Sham
B
Hippocampus
LF-MF
Sham
ntg.
n.ex
sAPPα
Tubulin
n.s.
sAPPα-level [%]
LF-MF
Sham
C
Cortex
LF-MF
Sham
ntg.
n.ex
sAPPβ
Tubulin
n.s.
sAPPβ-level [%]
LF-MF
Sham
D
Hippocampus
LF-MF
Sham
ntg.
n.ex
sAPPβ
Tubulin
n.s.
sAPPβ-level [%]
LF-MF
Sham

Suppl Fig. 2

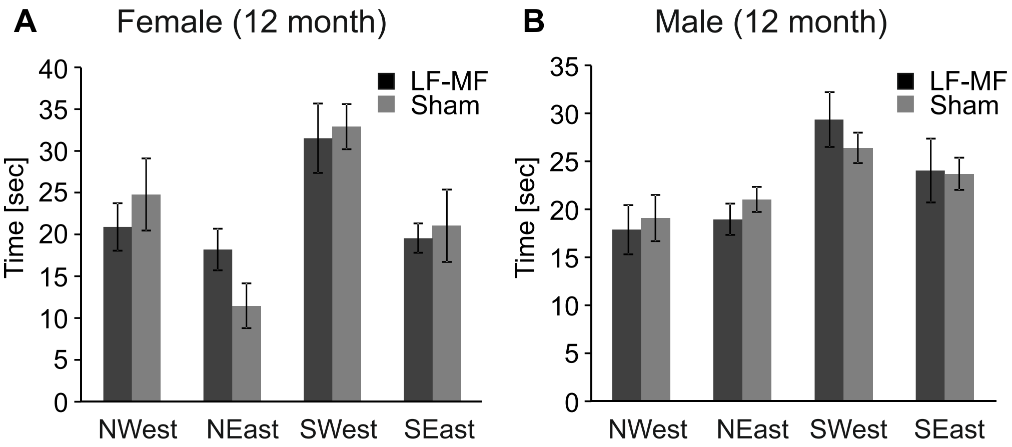
A
Female (12 month)
Time [sec]
40
35
30
25
20
15
10
5
0
LF-MF
Sham
NWest
NEast
SWest
SEast
B
Male (12 month)
Time [sec]
35
30
25
20
15
10
5
0
LF-MF
Sham
NWest
NEast
SWest
SEast

Suppl Fig. 3

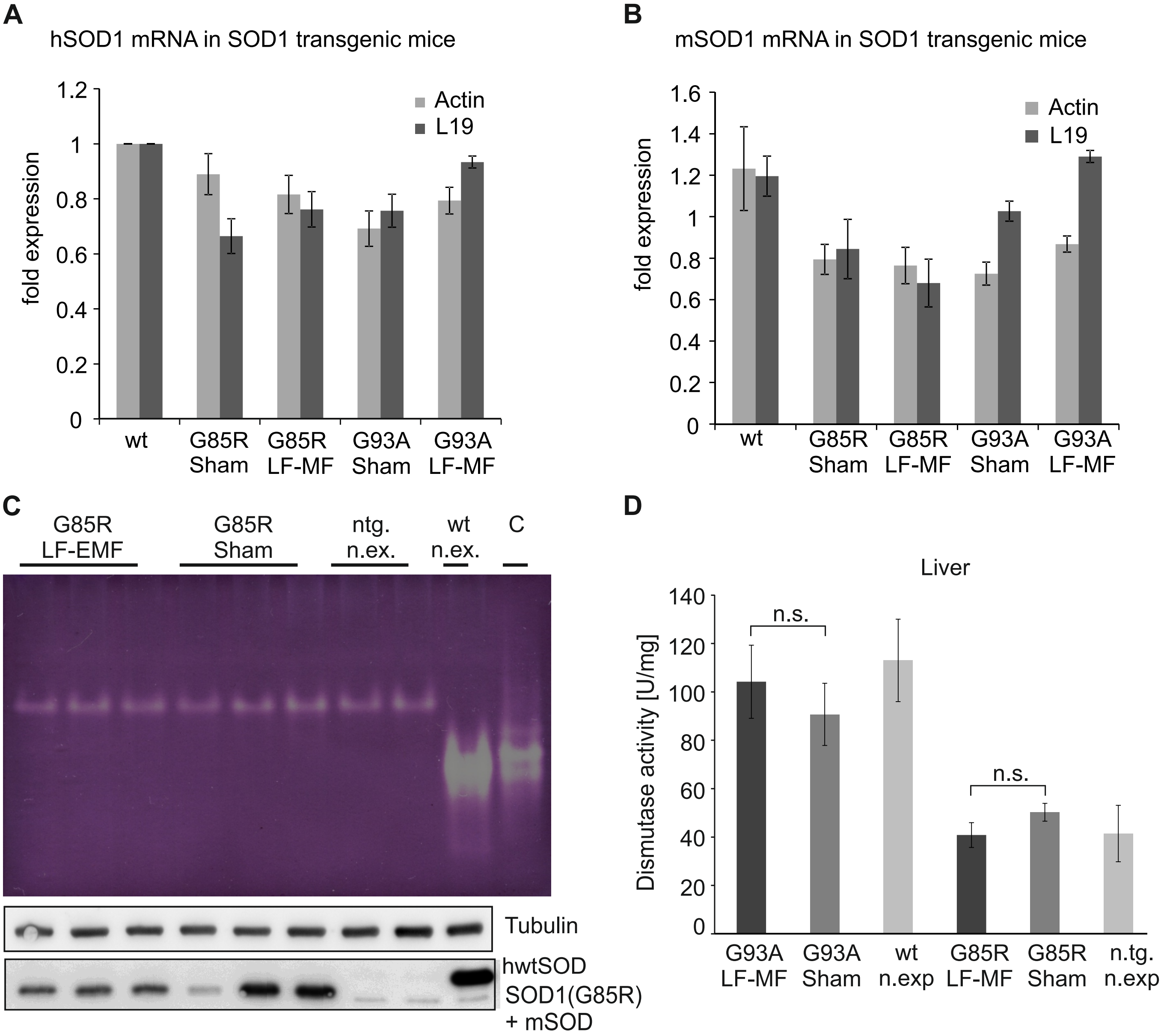
A
hSOD1 mRNA in SOD1 transgenic mice
fold expression
Actin
L19
wt
G85R Sham
G85R LF-MF
G93A Sham
G93A LF-MF
B
mSOD1 mRNA in SOD1 transgenic mice
fold expression
Actin
L19
wt
G85R Sham
G85R LF-MF
G93A Sham
G93A LF-MF
C
G85R LF-EMF
G85R Sham
ntg. n.ex.
wt n.ex.
C
Tubulin
hwtSOD
SOD1(G85R) + mSOD
D
Liver
Dismutase activity [U/mg]
n.s.
n.s.
G93A LF-MF
G93A Sham
wt n.exp
G85R LF-MF
G85R Sham
n.tg. n.exp

Suppl Fig. 4

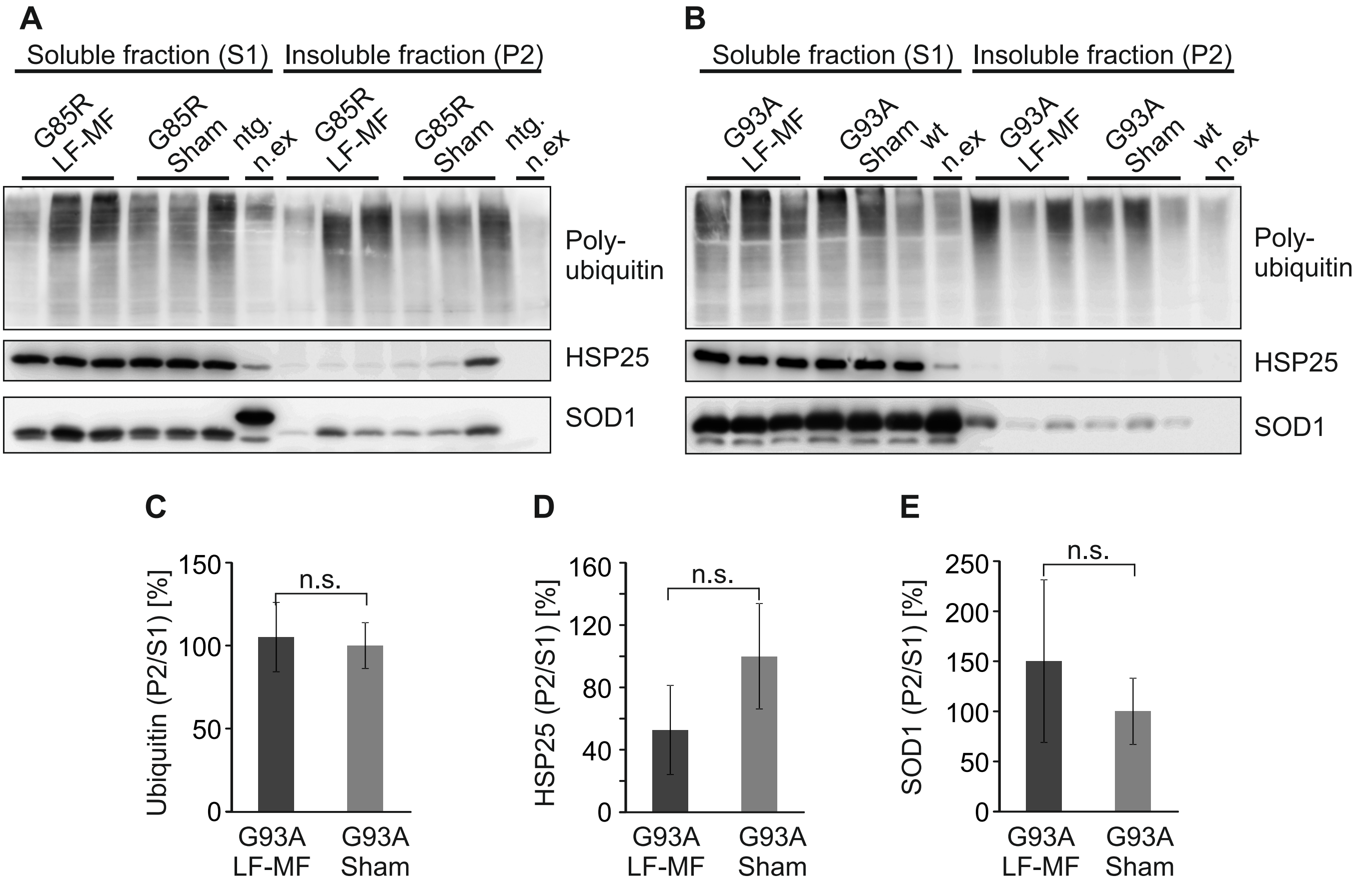

A
Soluble fraction (S1)
Insoluble fraction (P2)
G85R LF-MF
G85R Sham
ntg. n.ex
Poly-ubiquitin
HSP25
SOD1
B
Soluble fraction (S1)
Insoluble fraction (P2)
G93A LF-MF
G93A Sham
wt n.ex
Poly-ubiquitin
HSP25
SOD1
C
Ubiquitin (P2/S1) [%]
n.s.
G93A LF-MF
G93A Sham
D
HSP25 (P2/S1) [%]
n.s.
G93A LF-MF
G93A Sham
E
SOD1 (P2/S1) [%]
n.s.
G93A LF-MF
G93A Sham

Suppl. Fig. 5

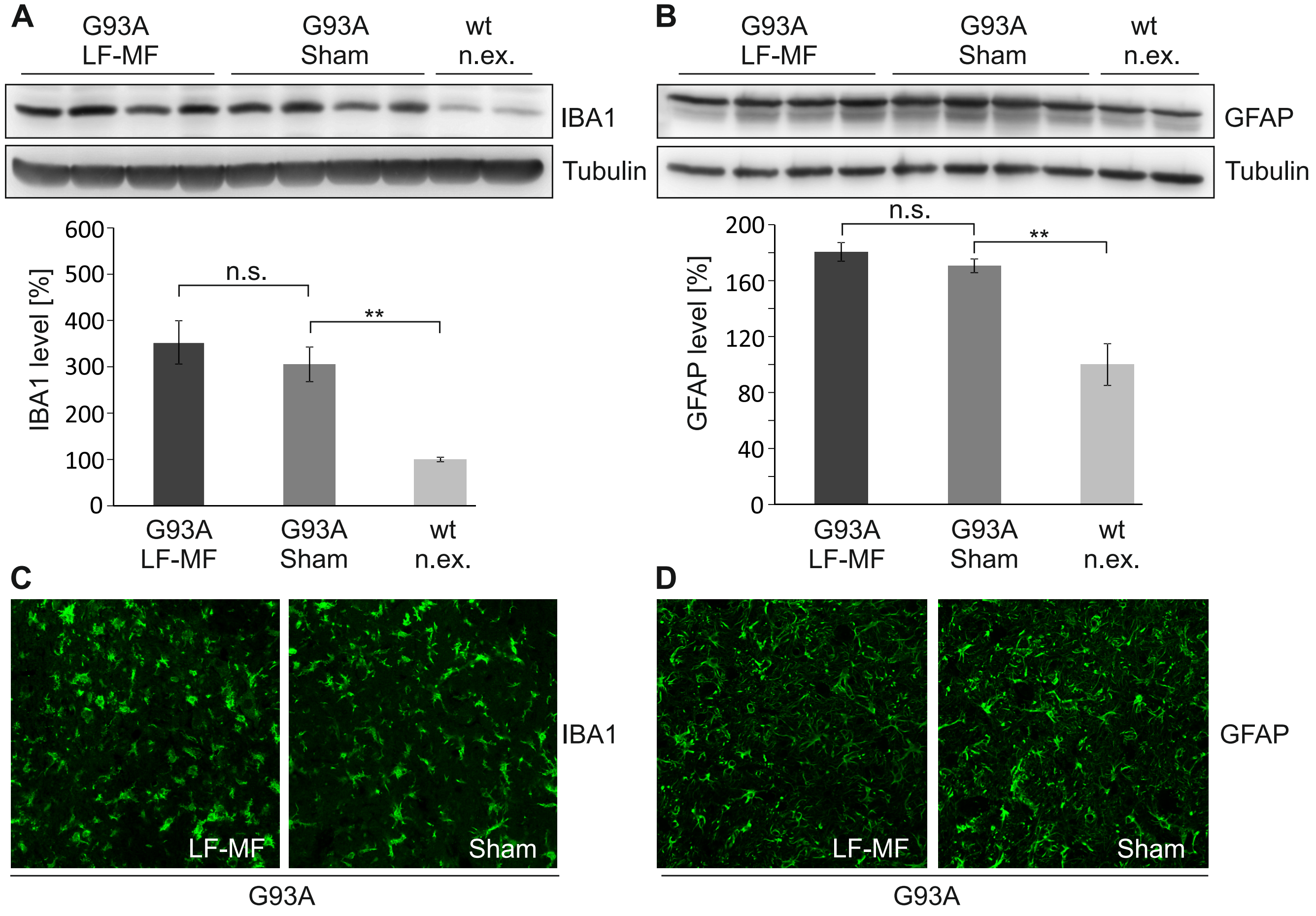
A
G93A LF-MF
G93A Sham
wt n.ex.
IBA1
Tubulin
IBA1 level [%]
600
500
400
300
200
100
0
n.s.
**
G93A LF-MF
G93A Sham
wt n.ex.
B
G93A LF-MF
G93A Sham
wt n.ex.
GFAP
Tubulin
GFAP level [%]
200
160
120
80
40
0
n.s.
**
G93A LF-MF
G93A Sham
wt n.ex.
C
IBA1
LF-MF
Sham
G93A
D
GFAP
LF-MF
Sham
G93A

Suppl Fig. 6

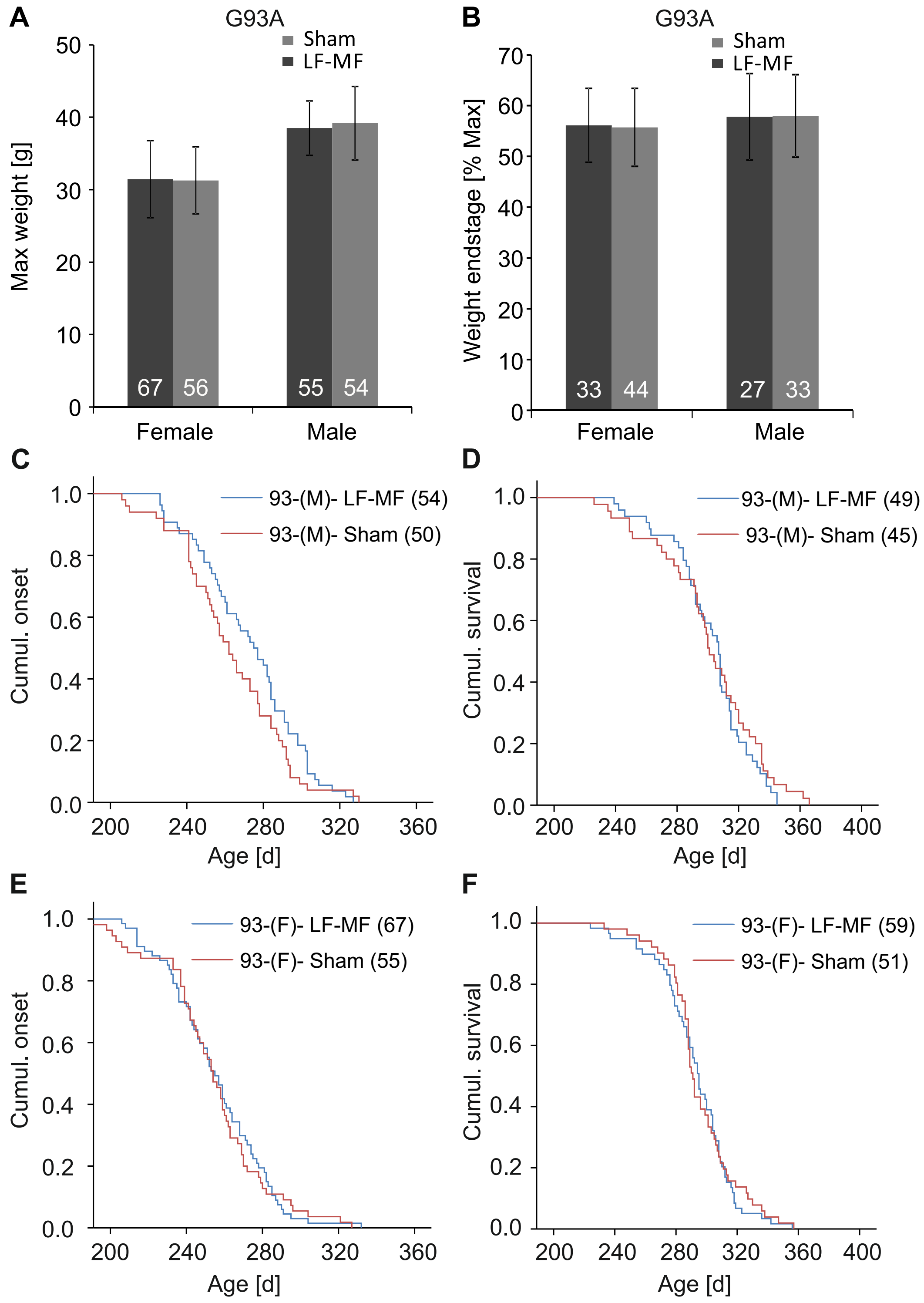
A
G93A
Sham
LF-MF
Max weight [g]
50
40
30
20
10
0
67
56
55
54
Female
Male
B
G93A
Sham
LF-MF
Weight endstage [% Max]
70
60
50
40
30
20
10
0
33
44
27
33
Female
Male
C
93-(M)- LF-MF (54)
93-(M)- Sham (50)
Cumul. onset
1.0
0.8
0.6
0.4
0.2
0.0
200
240
280
320
360
Age [d]
D
93-(M)- LF-MF (49)
93-(M)- Sham (45)
Cumul. survival
1.0
0.8
0.6
0.4
0.2
0.0
200
240
280
320
360
400
Age [d]
E
93-(F)- LF-MF (67)
93-(F)- Sham (55)
Cumul. onset
1.0
0.8
0.6
0.4
0.2
0.0
200
240
280
320
360
Age [d]
F
93-(F)- LF-MF (59)
93-(F)- Sham (51)
Cumul. survival
1.0
0.8
0.6
0.4
0.2
0.0
200
240
280
320
360
400
Age [d]

Suppl Fig. 7

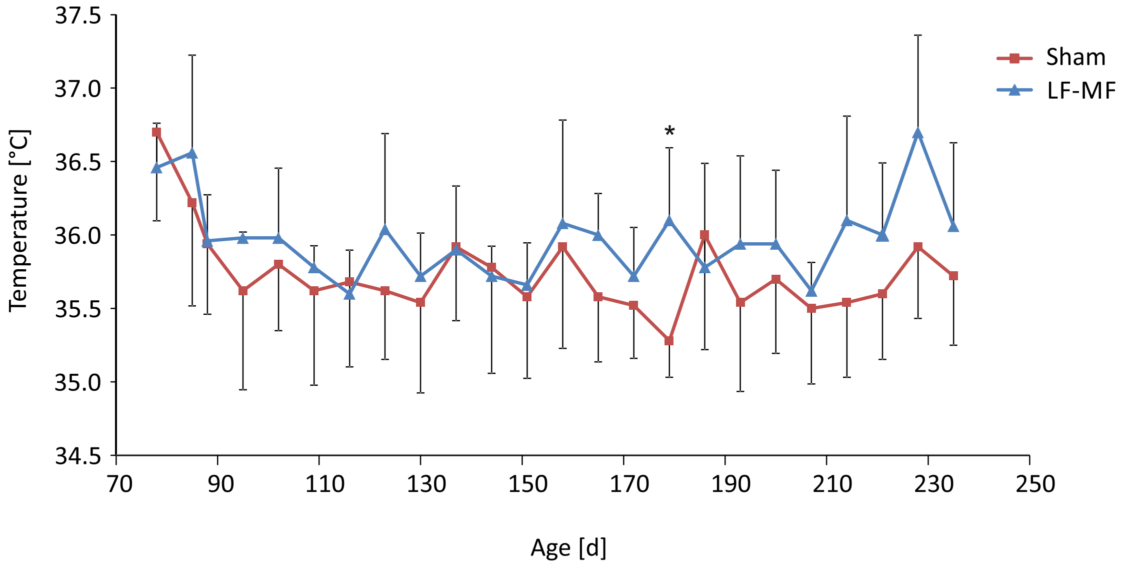

37.5
37.0
36.5
36.0
35.5
35.0
34.5
Temperature [°C]
70
90
110
130
150
170
190
210
230
250
Age [d]
Sham
LF-MF
*

Suppl Fig. 8

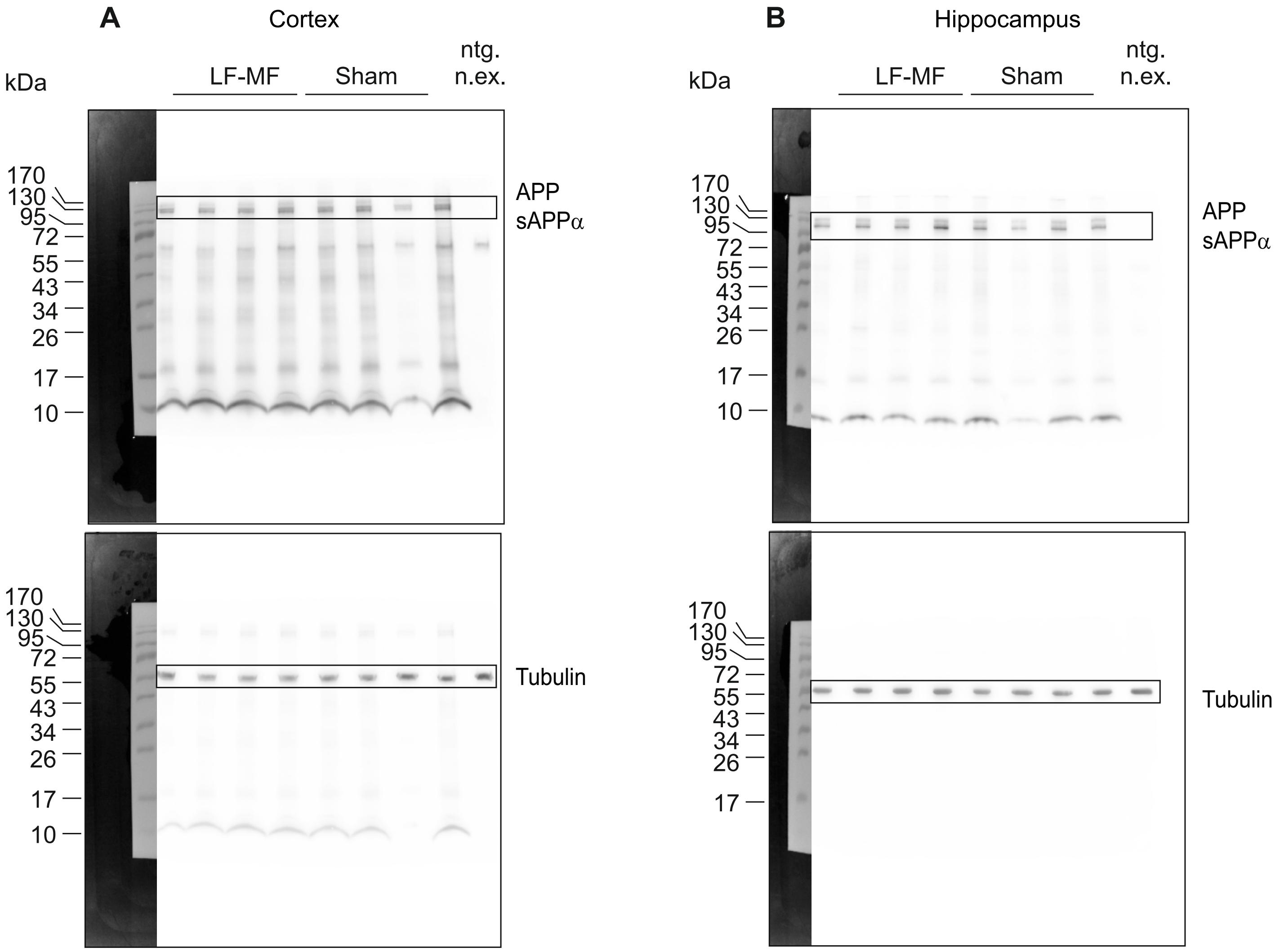
A
Cortex
kDa
LF-MF
Sham
ntg.
n.ex.
170
130
95
72
55
43
34
26
17
10
APP
sAPPα
Tubulin
B
Hippocampus

Suppl Fig. 9

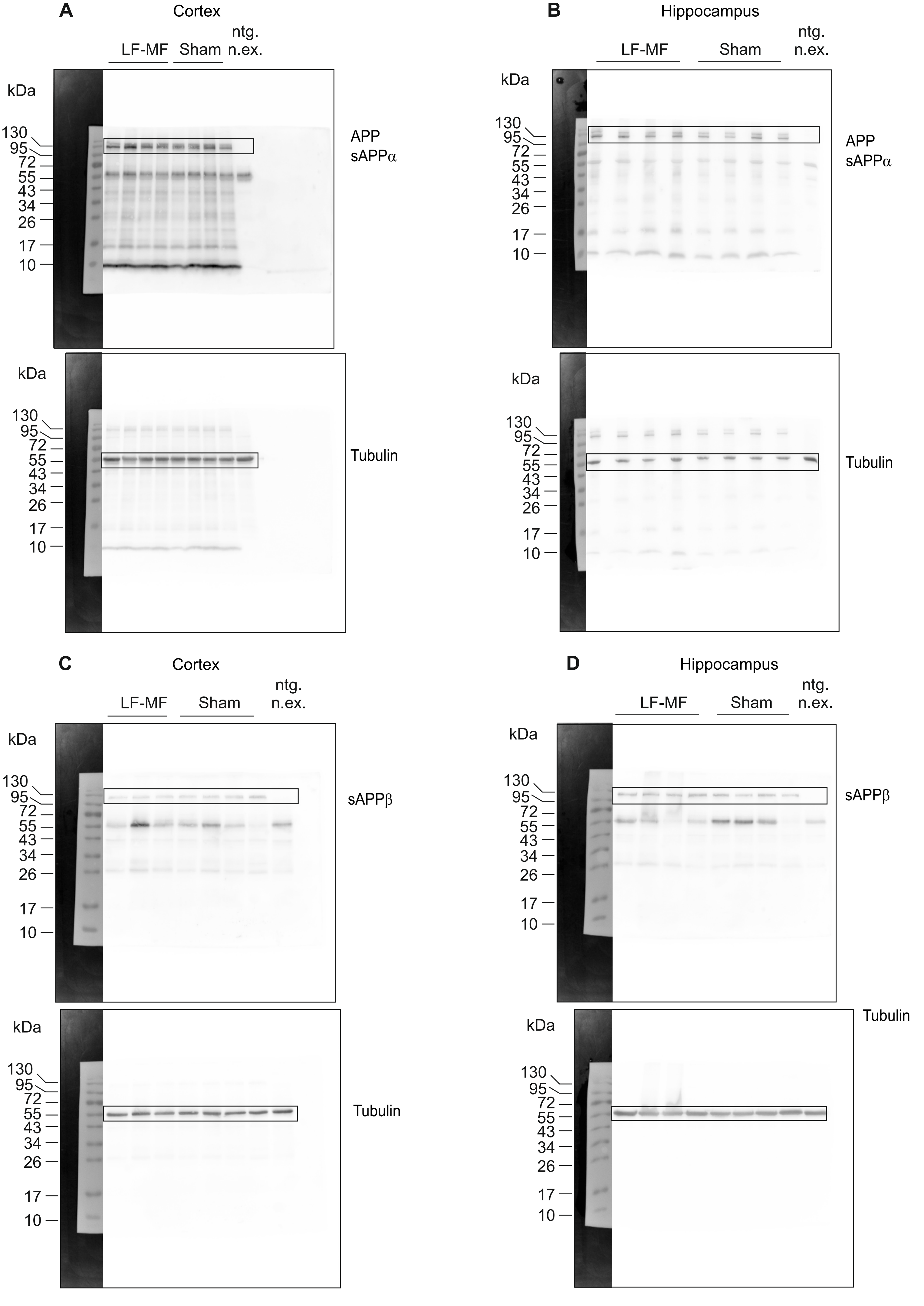
A
Cortex
LF-MF
Sham
ntg.
n.ex.
kDa
130
95
72
55
43
34
26
17
10
APP
sAPPα
Tubulin
B
Hippocampus
C
sAPPβ
D

Suppl Fig. 10

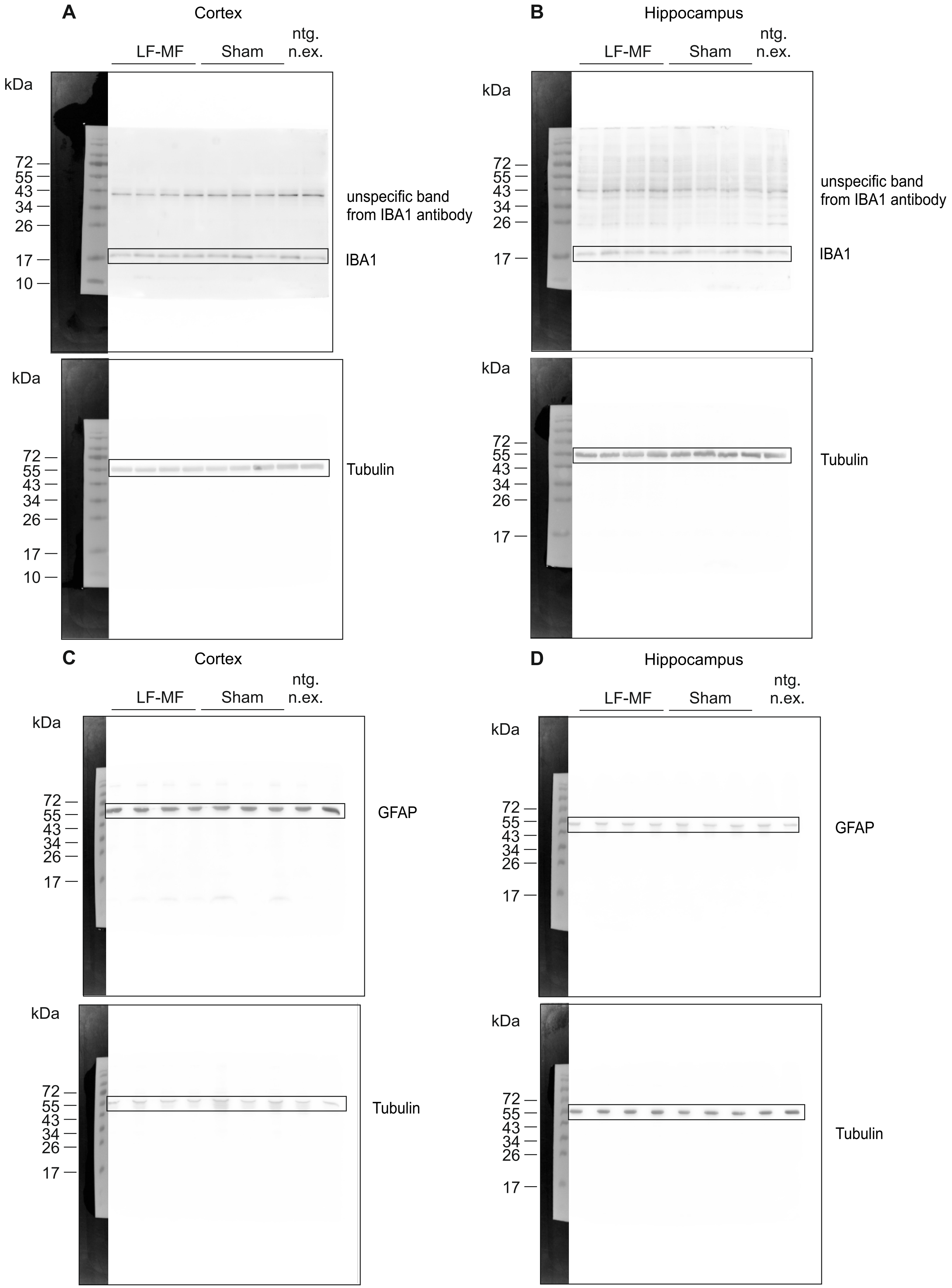
A
Cortex
LF-MF
Sham
ntg.
n.ex.
kDa
72
55
43
34
26
17
10
unspecific band
from IBA1 antibody
IBA1
Tubulin
B
Hippocampus
C
GFAP
D

Suppl Fig. 11

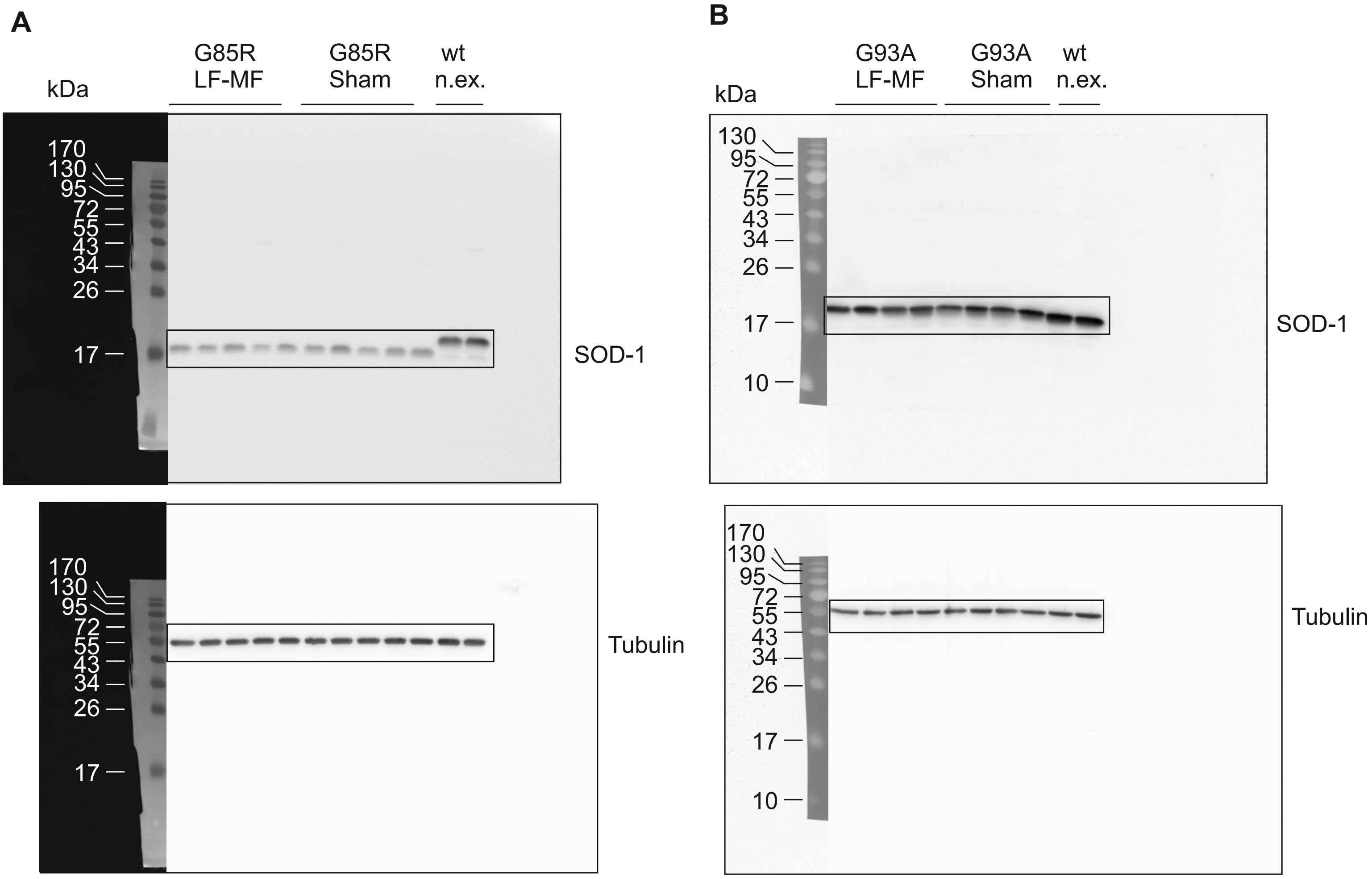
A
G85R
LF-MF
G85R
Sham
wt
n.ex.
kDa
170
130
95
72
55
43
34
26
17
SOD-1
Tubulin
B
G93A
LF-MF
G93A
Sham
wt
n.ex.
kDa
130
95
72
55
43
34
26
17
10
SOD-1
170
130
95
72
55
43
34
26
17
10
Tubulin

Suppl Fig. 12

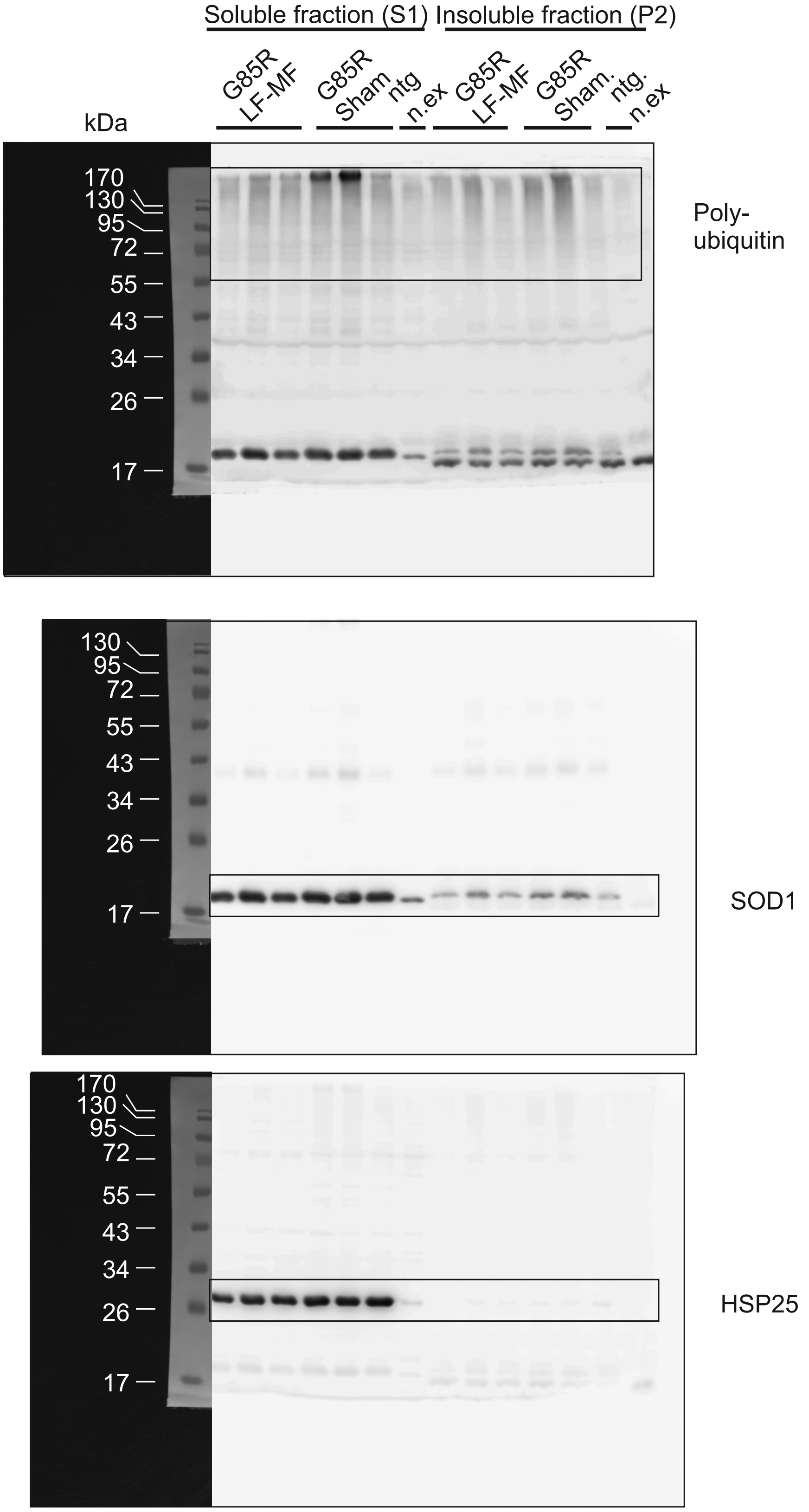
Soluble fraction (S1)
Insoluble fraction (P2)
G85R LF-MF
G85R Sham
ntg
n.ex
G85R LF-MF
G85R Sham.
ntg.
n.ex
kDa
170
130
95
72
55
43
34
26
17
Poly-ubiquitin
SOD1
HSP25

Suppl Fig. 13

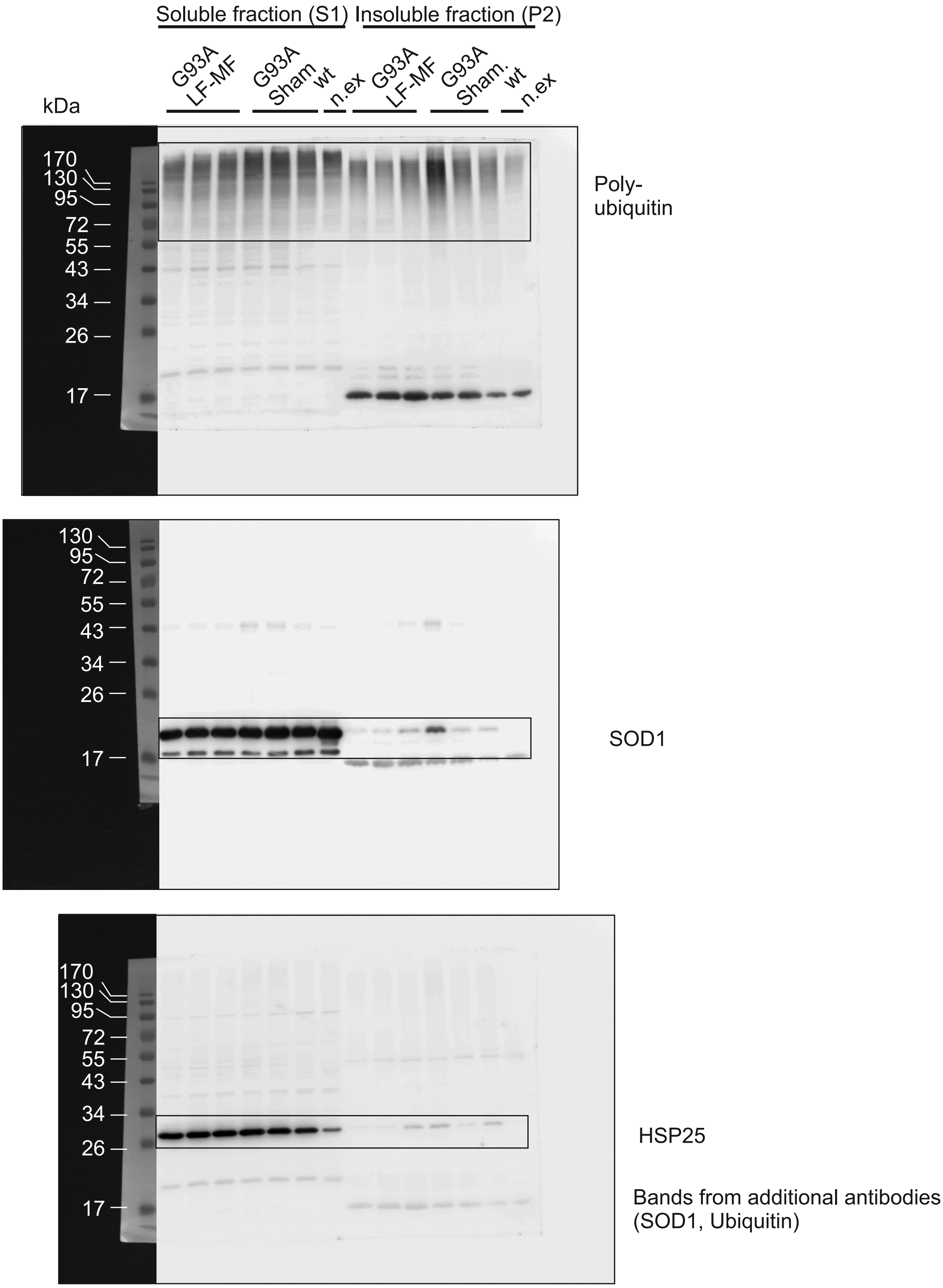
Soluble fraction (S1) Insoluble fraction (P2)
G93A LF-MF
G93A Sham
wt
n.ex
G93A LF-MF
G93A Sham.
wt
n.ex
kDa
170
130
95
72
55
43
34
26
17
Poly-ubiquitin
SOD1
HSP25
Bands from additional antibodies (SOD1, Ubiquitin)

Suppl Fig. 14

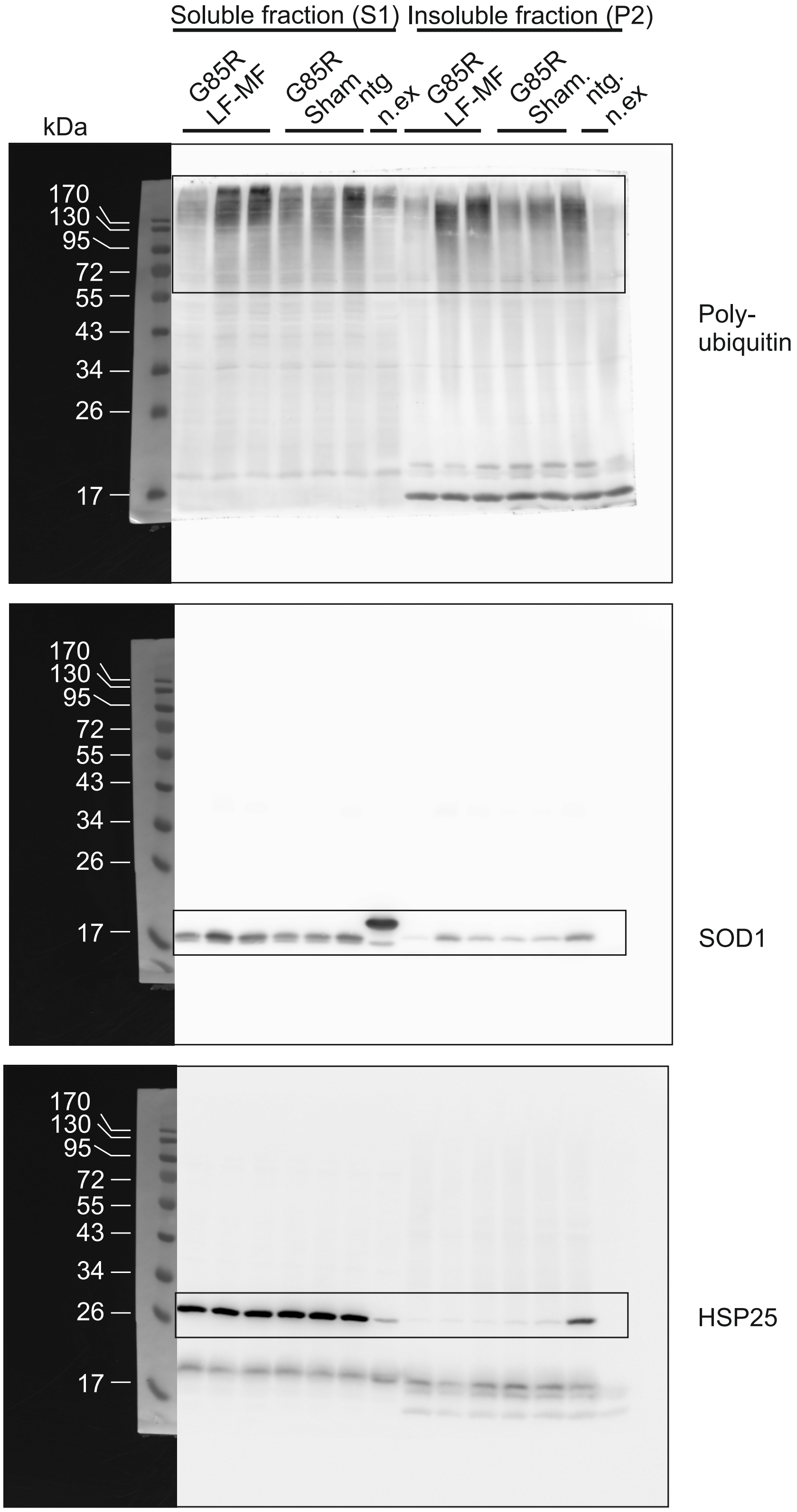
Soluble fraction (S1)
Insoluble fraction (P2)
G85R LF-MF
G85R Sham
ntg n.ex
G85R LF-MF
G85R Sham.
ntg. n.ex
kDa
170
130
95
72
55
43
34
26
17
Poly-ubiquitin
SOD1
HSP25

Suppl Fig. 15

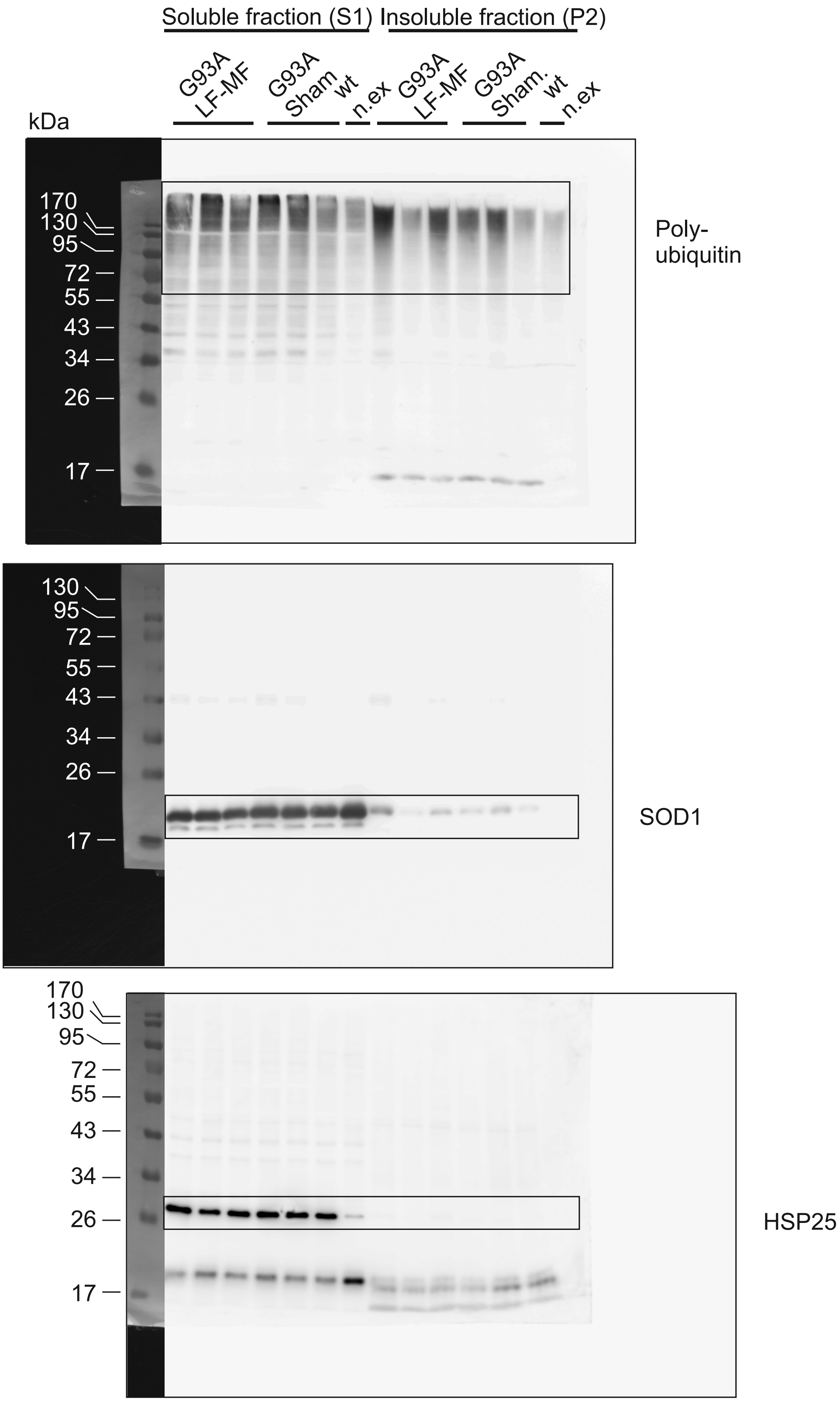
Soluble fraction (S1)
Insoluble fraction (P2)
G93A LF-MF
G93A Sham
wt
n.ex
G93A LF-MF
G93A Sham.
wt
n.ex
kDa
170
130
95
72
55
43
34
26
17
Poly-ubiquitin
130
95
72
55
43
34
26
17
SOD1
170
130
95
72
55
43
34
26
17
HSP25

Suppl Fig. 16

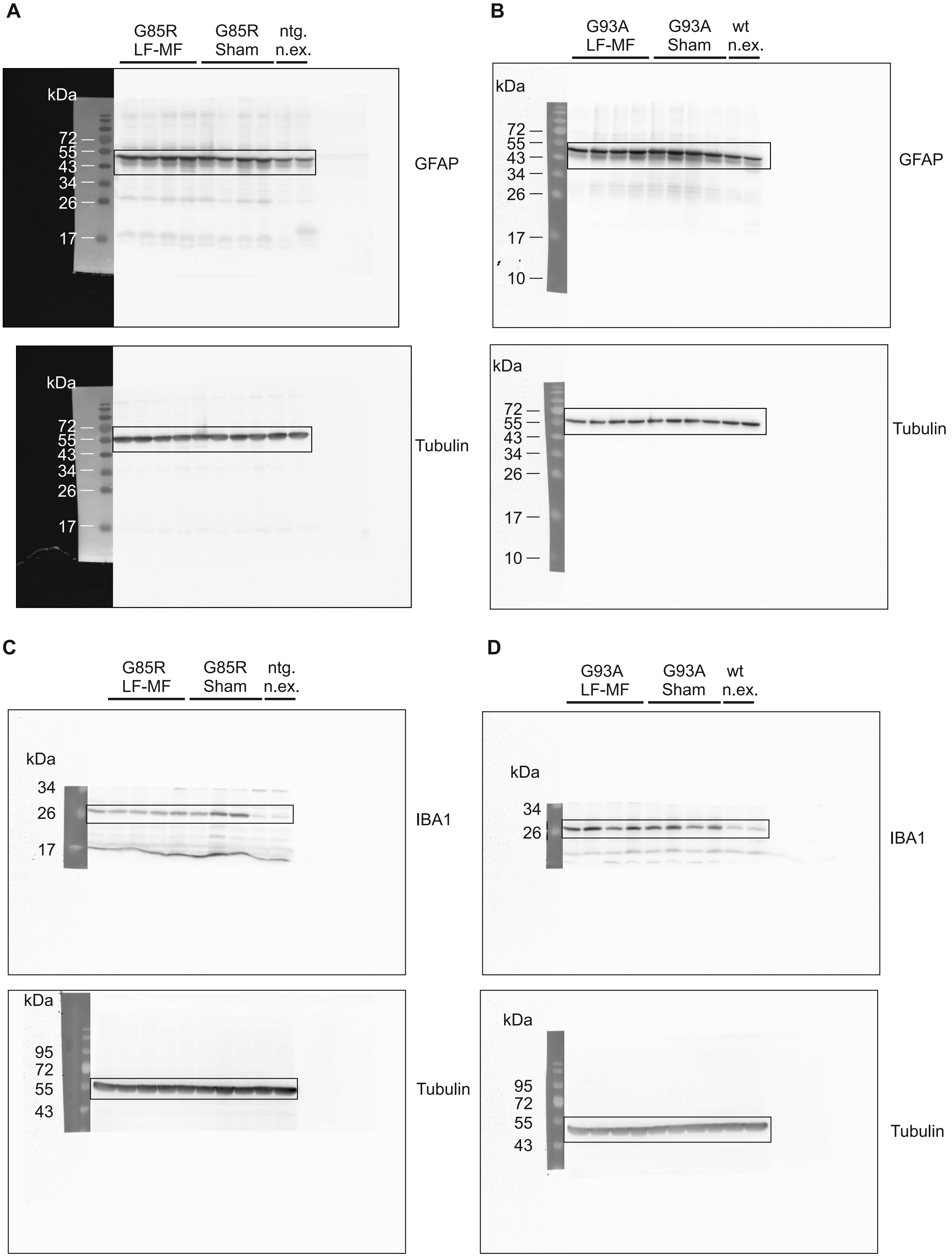
A
G85R LF-MF
G85R Sham
ntg. n.ex.
kDa
72
55
43
34
26
17
GFAP
Tubulin
B
G93A LF-MF
G93A Sham
wt n.ex.
kDa
72
55
43
34
26
17
10
GFAP
Tubulin
C
G85R LF-MF
G85R Sham
ntg. n.ex.
kDa
34
26
17
IBA1
95
72
55
43
Tubulin
D
G93A LF-MF
G93A Sham
wt n.ex.
kDa
34
26
IBA1
95
72
55
43
Tubulin

Suppl Fig. 17
